# Three Novel Bacteriophages for the Biocontrol of *Pseudomonas syringae* pv. *actinidiae* on Artificially Contaminated Kiwifruit Leaves

**DOI:** 10.3390/pathogens14121247

**Published:** 2025-12-05

**Authors:** Carla Pereira, Eduardo Gomes, Pedro Costa, João Duarte, Márcia Braz, Vanessa Oliveira, Newton C. M. Gomes, Victor M. Balcão, Adelaide Almeida

**Affiliations:** 1 Department of Biology and CESAM, University of Aveiro, Campus Universitário de Santiago, 3810-193 Aveiro, Portugal; eduardomfgomes@ua.pt (E.G.); pedrommrscosta@ua.pt (P.C.); j.macedoduarte@ua.pt (J.D.); marciabraz96@ua.pt (M.B.); v.oliveira@ua.pt (V.O.); gomesncm@ua.pt (N.C.M.G.); victor.balcao@prof.uniso.br (V.M.B.); 2 VBlab—Laboratory of Bacterial Viruses, University of Sorocaba, Sorocaba 18023-000, SP, Brazil

**Keywords:** phage biocontrol, *Pseudomonas syringae* pv. *actinidiae*, kiwifruit canker, sustainable bactericide

## Abstract

This study compared the efficacy of three individual lytic phages, PSA_LMAPSA-2T (PSA-2T), PSA_LMAPSA-6F (PSA-6F) and PSA_LMAPSA-7F (PSA-7F) and four phage cocktails (dual and triple combinations) in inactivating *Pseudomonas syringae* pv. *actinidiae.* Phages were isolated from kiwifruit leaves and soil samples contaminated with *P. syringae* pv. *actinidiae* and characterized by host spectrum, growth parameters, adsorption rate, genomic analysis, inactivation efficiency and viability under variable environmental conditions in orchard environments (temperature, pH and solar radiation). Phage PSA-2T showed the highest in vitro efficacy, achieving a 3.2 log CFU/mL maximum reduction after 18 h, outperforming PSA-6F and PSA-7F (0.6 and 1.5 log reductions, respectively). Phage cocktails achieved reductions of 1.0–2.2 log CFU/mL, but none exceeded the performance of PSA-2T alone. Phage viability was most affected by high temperature and acidic pH, with PSA-7F showing the greatest sensitivity. Nonetheless, all phages remained stable under typical orchard conditions. Phage PSA-2T significantly reduced *P. syringae* pv. *actinidiae* levels (1.5-log CFU/mL) on artificially contaminated kiwifruit leaves after a single treatment. These results demonstrate the potential of PSA-2T and phage cocktails as sustainable alternatives to copper and antibiotics, warranting further study of repeated treatments and broad-host-range phage formulations for field use.

## 1. Introduction

Kiwifruit production has steadily increased, reaching 4.5 million tons by 2022 [1]. The leading producers include China, New Zealand, Italy, Greece and Chile. However, this industry is severely affected by bacterial disease outbreaks, with devastating economic losses [2]. Among these, *P. syringae* pv. *actinidiae*, the causative agent of kiwifruit bacterial canker, is particularly destructive due to its rapid spread and high virulence [3]. *Pseudomonas syringae* pv. *actinidiae* comprises several biovars (biovar 1–6) that differ in genetics, virulence, and symptom expression [4,5,6]. Biovars 1 and 2 produce phytotoxins (phaseolotoxin and coronatine, respectively) and typically cause toxin-associated leaf spots [4,7]. Biovar 3, which does not produce known toxins, is the most aggressive and has been responsible for the major global pandemics affecting kiwifruit production [4,8]. Biovar 4, identified in New Zealand, is non-virulent and causes only mild leaf necrosis [3,4]. More recently, biovars 5 and 6 have been described in Japan; neither produces known toxins, except biovar 6, which can synthesize both phaseolotoxin and coronatine [4,6]. These biovars vary substantially in pathogenicity, ranging from non-virulent to highly destructive strains, making biovar identification essential for disease diagnosis, epidemiology, and management in kiwifruit orchards [4]. Infected plants exhibit a range of symptoms, often simultaneously, including flower darkening, necrotic leaf spots, discolored or darkened leaf veins, fruit blemishes, inhibition of seed germination, and stem cankers exuding bacterial ooze [9]. Different *P. syringae* pv. *actinidiae* biovars have been identified with varying virulence [5]. Despite ongoing efforts, controlling this pathogen remains extremely difficult, and infections frequently result in large-scale plant loss [10]. The economic impact has been significant. Following its emergence in Europe, the disease caused an estimated 2 million EUR in losses in Italy by 2009 and by 2014, damages in New Zealand were valued at approximately 930 million USD [11]. In response, the European Union (EU) introduced regulations to prevent the introduction and spread of *P. syringae* pv. *actinidiae* [12]. These include strict monitoring of all movements of *Actinidia* spp. (*Actinidiaceae* family) plants, fruits, and pollen, alongside origin traceability and mandatory annual surveys [13]. However, by 2020, a Pest Survey Card issued by the European Food Safety Authority confirmed the presence of *P. syringae* pv. *actinidiae* in several kiwifruit-growing areas across the EU [14]. As a result, more stringent measures were implemented, such as increasing the radius for plant destruction around detected outbreaks from 5 to 500 m [13]. However, despite these efforts, the pathogen continues to spread, with considerable negative effects on production.

Currently, kiwifruit growers rely mostly on preventive measures, as there is no effective method to eradicate *P. syringae* pv. *actinidiae*. Copper-based products have long been used in fruit production to control fungal and bacterial diseases. Common formulations include copper sulfate, copper oxychloride, and basic copper salts. In Europe, copper-based products are widely used [4,15]. However, due to copper’s persistence and accumulation in soil, which can harm soil and aquatic ecosystems, its use has been strictly limited or completely banned in some regions for over 20 years [16]. These restrictions have promoted integrated management strategies, including resistant cultivars, cultural practices, precision application, and alternative organic-approved products [4,16]. Streptomycin is used in New Zealand and several Asian countries to manage plant bacterial diseases, and trunk injections in Korea have shown promising results for kiwifruit protection [2,4,15,17,18]. However, its use is prohibited in many European countries, including Italy and Portugal, due to concerns about inducing bacterial resistance and leaving residues on fruit [2,4]. However, prolonged copper use can lead to soil toxicity, disrupt microbial communities [19] and contribute to antimicrobial resistance. Copper can also bioaccumulate in plants and contaminate nearby watercourses through agricultural runoff, impacting surrounding ecosystems. Although antibiotics are employed in some countries, the rise of antibiotic-resistant bacteria is a growing global concern [4]. Furthermore, their use is increasingly restricted due to issues such as residue accumulation in leaves and fruits, environmental pollution, and changes in soil bacterial communities [20,21]. Consequently, the use of streptomycin has been banned in certain European countries, including Portugal [4]. Given these limitations, there is a pressing need for alternative, sustainable strategies that minimize the risk of developing resistant *P. syringae* pv. *actinidiae* strains. In recent years, innovative approaches have been explored, including antimicrobial peptides, microbial biocontrol agents (fungi and bacteria), bioactive compounds, antimicrobial photodynamic therapy, and bacteriophage-based treatments [4,22].

Phages present unique advantages over conventional antibacterial applications. Their specificity for target bacteria minimizes disruption to native soil microbial communities [23], thereby reducing environmental concerns such as contamination from agricultural runoff. Phages infect bacteria through interactions between their attachment structures and bacterial surface receptors. Once a phage adsorbs to its host, the infection can proceed through one of two major life cycles: lysogenic or lytic [24,25]. In the lysogenic cycle, temperate phages integrate their genome into the bacterial chromosome or persist as extrachromosomal elements, establishing a stable association with the host [24]. During this state, the phage does not immediately destroy the cell and can instead influence bacterial traits, including virulence, through horizontal gene transfer. Because lysogeny may enhance pathogenicity or transfer unwanted genes, temperate phages are unsuitable for therapeutic applications. In contrast, lytic phages initiate a strictly destructive infection cycle. After attachment and genome injection, they rapidly take over host metabolism, replicate their components, assemble new virions, and ultimately lyse the bacterial cell aided by holins and endolysins, releasing progeny phages [24,26,27]. This ability to specifically target and kill bacteria makes lytic phages the preferred choice for phage therapy and biocontrol strategies [24,25]. Moreover, phages can penetrate plant tissues and reach internal infection sites, enhancing disease control [28].

One major limitation of phage-based biocontrol, however, is their sensitivity to ultraviolet (UV) radiation [23,29,30,31,32]. This problem can be overcome by applying phages during low-light periods or using protective carriers, such as nanoparticles, to shield them from UV damage [31]. Iriarte and his coworkers (2012) studied phage survival rates in plant tissues and detected active phages in tomato plant roots, stems and leaves up to 15 days post-inoculation [33]. Furthermore, phage replication within bacterial hosts can sustain high viral concentrations at infection sites [34,35]. Despite these promising features, no phage-based treatments are currently approved for the control of *P. syringae* pv. *actinidiae*-induced kiwifruit canker [23,24]. Nonetheless, several research groups have reported on the isolation, morphological characterization and genome sequence of phages with potential activity against *P. syringae* pv. *actinidiae* [32,35,36,37,38,39,40,41,42,43,44]. Promising results have also been obtained in ex vivo studies [30,32,35] and in whole-plant assays where phage applications reduced *P. syringae* pv. *actinidiae* infections without harming host plants [35,36,43,44]. Our research group previously demonstrated the potential of the commercially available phage ϕ6 in controlling *P. syringae* pv. *actinidiae* in vitro and ex vivo, suggesting its applicability in kiwifruit orchards [30]. However, this field remains underexplored [4]. To develop effective phage-based biocontrol strategies, further in planta studies are needed—particularly those involving phage cocktails with broad host ranges capable of targeting diverse *P. syringae* pv. *actinidiae* strains under variable environmental conditions. Despite ongoing isolation efforts, few phage cocktails are currently available for field application in kiwifruit orchards [4]. In this study, we report the isolation and characterization of three novel lytic phages active against *P. syringae* pv. *actinidiae*. Their biocontrol potential was assessed through in vitro and ex vivo experiments, contributing to the development of phage-based strategies for sustainable management of bacterial canker in kiwifruit.

## 2. Materials and Methods

### 2.1. Bacterial Strain and Growth Conditions

*Pseudomonas syringae* pv. *actinidiae* strain CRA-FRU 14.10, a biovar 3 isolate [30,45] representing the highly virulent lineage responsible for recent global kiwifruit outbreaks, was used as the host for phage propagation in this study. The bacterial strains used for the host range experiment are listed in Table 1. *Aeromonas hydrophila* (ATCC 7966), *Escherichia coli* (ATCC 25922 and ATCC 13706), *Pseudomonas aeruginosa* (ATCC 27853), *Salmonella enterica* serovar Typhimurium (ATCC 14028 and ATCC 13311), *P. syringae* pv. *syringae* (DSM 21482), *Staphylococcus aureus* (DSM 25693), and *Vibrio parahaemolyticus* (DSM 27657) were purchased from ATCC and DSM culture collections. *P. syringae* pv. *actinidiae* strains CRA-FRU 8.43, 12.54 and 14.10 were obtained from the Culture Collection of Centro di Ricerca per la Frutticoltura (CRA-FRU; Rome, Italy) [45,46]. Additional *Pseudomonas* strains were previously isolated from water samples in Ria de Aveiro, Portugal [47,48].

All bacterial strains were preserved at −80 °C in Tryptic Soy Broth medium (TSB, Liofilchem, Roseto degli Abruzzi, Italy) supplemented with 25% (*v*/*v*) glycerol and subcultured only once before use. Working plates were maintained at 4 °C for no longer than 48 h. Before each assay, stock cultures were aseptically inoculated into 30 mL of TSB and incubated for 16 h at 25 °C with orbital shaking (120 rpm). A 300 µL aliquot was then transferred to fresh TSB (30 mL) and grown under the same conditions for 16 h.

### 2.2. Phage Isolation and Purification

Phages PSA-6F and PSA-7F were isolated from *P. syringae* pv. *actinidiae*-infected kiwifruit leaves, and phage PSA-2T from kiwifruit orchard soil in Aveiro (Portugal), using *P. syringae* pv. *actinidiae* CRA-FRU 14.10 as the host. For isolation, 10 g of leaf or soil material was homogenized in 40 mL of deionized water and incubated at 25 °C for 2 h at 120 rpm. Following centrifugation (10,000× *g*, 10 min, 4 °C), the supernatant was filtered through a 0.22 µm polyethersulfone (PES) membrane (Merck-Millipore, Darmstadt, Germany). To enrich for phages, 10 mL of the filtrate was mixed with 90 mL of double-strength TSB and inoculated with 500 µL of an overnight host culture. The mixture was incubated at 25 °C for 48 h under gentle shaking (80 rpm), then centrifuged and filtered as before. Phage concentrations were determined using the double-layer agar method [49], with Tryptic Soy Agar (TSA, Liofilchem, Roseto degli Abruzzi, Italy) as the culture medium. Plates were incubated at 25 °C and examined for lytic plaques after 24 h. A single plaque was picked and inoculated into TSB containing a fresh host culture. After incubation and centrifugation, the supernatant was used for subsequent rounds of plaque purification. Three successive single-plaque isolation cycles were performed to obtain pure phage stocks.

Phage stocks were prepared in SM buffer (0.1 M NaCl [Honeywell Fluka™; Seelze, Germany], 20 mM Tris-HCl [Sigma; St. Louis, MO, USA], and 8 mM MgSO_4_, pH 7.5), using the host strain CRA-FRU 14.10. Exponentially growing bacterial cultures were centrifuged (10,000× *g*, 10 min), and the pellets were resuspended in 50 mL of SM buffer. Subsequently, 500 µL of purified phage suspension was added and incubated at 25 °C for 24 h under gentle orbital shaking (50 rpm). Lysates were centrifuged and filtered (0.22 µm), and phage titres were determined by the double-layer agar method [49]. Plaques were counted after 24 h of incubation at 25 °C, and titres were expressed as plaque-forming units (PFU)/mL. Final phage suspensions were kept at 4 °C.

### 2.3. Virion Morphology

Phage particles from highly concentrated suspensions (109 PFU/mL) were negatively stained with 2% (*w*/*w*) uranyl acetate (Electron Microscopy Sciences; Hatfield, UK) for 1 min. Electron photomicrographs were captured using a JEOL 1011 transmission electron microscope (TEM) (JEDLUSA Inc; Peabody, MA, USA) at 100 kV, equipped with a high-resolution CCD camera (model CCD-Erlangshen ES100W) (GATAN Inc.; Pleasanton, CA, USA).

### 2.4. SDS-PAGE Analysis

Phage suspensions were purified using the polyethylene glycol (PEG) precipitation method [50]. Each suspension was mixed with a sterile solution of PEG 8000 (10%, *w*/*w*; Sigma-Aldrich; St. Louis, MO, USA) and NaCl (1 M; Sigma-Aldrich; St. Louis, MO, USA) at a 2:1 (*v*/*v*) ratio. The mixtures were incubated overnight at 4 °C and centrifuged at 14,880× *g* for 45 min at 4 °C. The supernatant was discarded, and the resulting pellets were resuspended and homogenized in 5 mM MgSO_4_ (Sigma-Aldrich; St. Louis, MO, USA). To further concentrate the phage particles, 500 µL of the PEG-precipitated suspension was transferred to Amicon^®^ Ultra centrifugal filters (Ultracel^®^-10k, regenerated cellulose, 10,000 NMWL; Merck Millipore Ltd., Tullagreen, Cork, Ireland) and centrifuged at 17,709× *g* for 6 min. The filter unit was then inverted into clean Eppendorf^®^ tubes and centrifuged again under the same conditions to recover the retentate, which was used for SDS–PAGE analysis. Structural protein profiles of the purified phage particles were analyzed by sodium dodecyl sulfate–polyacrylamide gel electrophoresis (SDS–PAGE) using a Mini-PROTEAN^®^ Tetra Cell system (Bio-Rad, Hercules, CA, USA) connected to a PowerPac™ HC power supply and a Digital Dry Bath (Bio-Rad). For sample preparation, 500 µL of each phage suspension was mixed with 500 µL of disruption buffer (Bio-Rad, Santo Amaro, SP, Brazil) containing 1.51% (*w*/*v*) Tris-base, 0.5% (*v*/*v*) β-mercaptoethanol, 4% (*w*/*v*) SDS, 10% (*v*/*v*) glycerol, and 0.012% (*w*/*v*) bromophenol blue. Samples were heated at 95 °C for 10 min before electrophoresis. A total of 20 µL of each sample and 5 µL of molecular weight markers (Pre-stained Precision Plus Protein™ Dual Color Standards, Bio-Rad; range: 20–250 kDa) were loaded onto gels consisting of a 5% stacking gel and a 12% resolving gel. Electrophoresis was performed at 200 V (20 mA per gel; 20 W) for 60 min. Gels were stained with Coomassie Brilliant Blue R-250 and imaged using the Gel Doc™ EZ System (Bio-Rad, CA, USA).

### 2.5. Determination of the Phage Host Range and Efficiency of Plating (EOP)

The host range of the phages was determined by spot testing following previously published protocols [49]. Briefly, 10 µL of each phage suspension was spotted onto bacterial lawns prepared on double-layer agar plates. Following incubation at 25 °C for 24 h, susceptibility was recorded as the presence (+) or absence (−) of a clear lysis zone. For bacterial strains exhibiting lysis, the EOP was calculated as the ratio between the phage titre (PFU/mL) obtained on the test strain and that obtained on the original host strain [51]. Plaque counts were determined using the double-layer agar method [49], and EOP values were calculated from three independent measurements.

### 2.6. Determination of the Adsorption Curves

Phage adsorption assays were performed as previously described [29]. Phages were added to exponentially growing host cultures in TSB medium at 25 °C. Aliquots were collected every 20 min, treated with a few drops of chloroform, centrifuged (10,000× *g*, 5 min, 4 °C), and the supernatants were filtered through 0.22 µm membranes. The number of unadsorbed phage particles was determined by the double-layer agar method [49]. Phage adsorption was expressed as the percentage decrease in phage titre relative to the initial value (time zero). Control assays without bacterial cells were included as no-adsorption references [52]. All experiments were conducted in triplicate.

To estimate adsorption and desorption kinetics, data were fitted to an exponential decay model assuming reversible binding of phage virions to susceptible bacterial cells:

$$\frac{P_{t}}{P_{0}}=\frac{\phi }{\delta \cdot X_{0}+\phi }\left\{1+\frac{\delta \cdot X_{0}}{\phi }\cdot e^{-\left(\delta \cdot X_{0}+\phi \right)\cdot t}\right\}$$

where *P_t_* and *P*_0_ represent phage concentrations (PFU/mL) at time *t* and at time zero, respectively; δ is the first-order adsorption rate constant (mL CFU^−1^ min^−1^); ϕ is the first-order desorption rate constant (min^−1^); *X*_0_ denotes the initial concentration of susceptible bacterial cells (CFU/mL); and *t* is the infection time (min). Model fitting was performed by nonlinear regression using the Solver function in Microsoft Excel for Mac (Version 16.103.3 (25113013)) (Microsoft, Redmond, WA, USA), yielding estimates of both adsorption and desorption parameters [50].

### 2.7. Determination of the One-Step Growth Curves

Phage suspensions were mixed with 10 mL of host culture to achieve a multiplicity of infection (MOI) of 0.001. The mixtures were incubated for 5 min at 25 °C without agitation, then centrifuged (10,000× *g*, 5 min). The supernatants were discarded, and the pellets were resuspended in 10 mL of TSB. Incubation continued at 25 °C following the method of [53]. Aliquots (1 mL) were collected at time zero and at 20-min intervals, immediately plated using the double-layer agar method [49], and incubated at 25 °C for 24 h. Phage growth kinetics were analyzed based on one-step growth curves as described by [29]. Three independent experiments were performed.

### 2.8. Bacterial Inactivation Assays Using Single Phage Suspensions and Phage Cocktails

Bacterial inactivation was evaluated using individual phages and phage cocktails composed of two or all three phages in equal proportions, against *P. syringae* pv. *actinidiae* CRA-FRU 14.10 at an MOI of 1. Exponential-phase bacterial cultures (OD_600_ = 0.7; approximately 10^8^ CFU/mL) were diluted to 10^5^ CFU/mL and mixed with phages (10^5^ PFU/mL) in 30 mL of TSB. Cultures were incubated at 25 °C without agitation. Two controls were included: a bacterial control (BC; bacteria without phage) and a phage control (PC; phage without bacteria). Samples were collected at 0, 6, 12, 18, and 24 h. Phage titres were determined using the double-layer agar method [49] after 24 h incubation at 25 °C, and bacterial concentrations were assessed by the drop-plate method after 18 h incubation at 25 °C [30]. All assays were performed in triplicate across three independent experiments.

### 2.9. Determination of the Rate of Emergence of Phage-Resistant Bacterial Mutants

The emergence of *P. syringae* pv. *actinidiae* CRA-FRU 14.10 mutants resistant to individual phages or phage cocktails were assessed following the procedure of [54]. Ten individual colonies of susceptible bacteria were transferred from TSA plates into separate tubes containing 5 mL of TSB and incubated at 25 °C for 24 h, reaching approximately 10^9^ CFU/mL. Aliquots of serially diluted bacterial cultures (10^0^ to 10^−3^) were mixed with 0.1 mL of phage suspension (10^9^ PFU/mL) in molten TSA to achieve an MOI of 1. The mixtures were spread onto TSA plates and incubated at 25 °C for 3–5 days to allow the growth of potential phage-resistant mutants. This incubation period corresponds to multiple complete lytic cycles of the phages, ensuring that colonies appearing on these plates represent true resistant mutants rather than bacteria that had not yet undergone phage infection. Bacterial cells acquire resistance to the phage after 3–5 days following contact with the phage virions, and therefore, new infection cycles begin.

In parallel, 0.1 mL aliquots from bacterial dilutions (10^−5^ to 10^−7^) were plated without phage and incubated for 24 h at 25 °C to estimate the total number of phage-sensitive cells. The rate of emergence of resistant mutants was calculated as the ratio of the number of resistant colonies (from phage-treated plates) to the total number of sensitive bacteria (from control plates). All assays were performed in triplicate in three independent experiments.

### 2.10. Determination of the Impact of Environmental Factors on Phage Viability

The effects of temperature, pH, and solar radiation on the viability of the phages (10^7^ PFU/mL) were evaluated in 30 mL of sterile phosphate-buffered saline (PBS). For temperature stability, samples were incubated at 17, 25, and 40 °C (pH 7.0). For pH stability, samples were adjusted to pH 5.0, 7.0, and 10.0 (25 °C). Solar radiation assays were conducted at pH 7.0 under natural sunlight, with control samples (SR-C) maintained under identical conditions but shielded from direct exposure. The experiments were performed on a day with a mean solar irradiance of 6.92 kWh/m^2^ and ambient temperatures ranging from 12 to 25 °C. Phage titres were determined at 0, 24, 48, and 72 h for temperature and pH conditions, and at 0, 1, 2, 3, 4, 5, and 6 h for solar exposure. Quantification was performed using the double-layer agar method in triplicate after 18 h incubation at 25 °C [30]. Each condition was tested in three independent experiments.

### 2.11. Determination of Bacterial Inactivation by Phage PSA-2T

The optimal in vitro conditions for phage PSA-2T treatment (inactivation rate and incubation time) were evaluated at MOIs 1, 10, and 100 against the bacterial host. Each assay included two controls: a bacterial control (BC; bacteria without phage) and a phage control (PC; phage without bacteria). Test samples (BP; bacteria plus phage) and controls were incubated under identical conditions, and aliquots were collected at 0, 6, 12, 18, and 24 h. Phage titres were determined in triplicate by the double-layer agar method after 24 h incubation at 25 °C [30]. Bacterial concentrations were quantified in triplicate using the drop-plate method on TSA. Each condition was tested in three independent experiments.

### 2.12. Determination of Bacterial Inactivation by Phage Treatment on Kiwifruit Plant Leaves Artificially Contaminated with P. syringae pv. actinidiae

The efficacy of phage PSA-2T against *P. syringae* pv. *actinidiae* CRA-FRU 14.10 was assessed on kiwifruit (*Actinidia deliciosa*) leaves under controlled ex planta conditions. Leaves without visible *P. syringae* pv. *actinidiae* symptoms were collected from a kiwifruit orchard in Aveiro, Portugal, and stored at 4 °C until use. Prior to assays, leaves were labelled, cut into 3 × 3 cm squares, and surface-sterilized by immersion in 3% (*v*/*v*) H_2_O_2_ for 15 min, followed by UV-C irradiation (5.5 W) on both sides for 10 min within a sterilizing chamber [55].

Six experimental groups were prepared, each comprising three replicates of four leaves (72 samples in total). Three groups were inoculated with *P. syringae* pv. *actinidiae* CRA-FRU 14.10 (10^5^ CFU/mL): two were treated with phage PSA-2T at MOI 10 and MOI 100, and one served as the bacterial control (BC). Two groups were treated with phage PSA-2T alone (phage controls, PC-10 and PC-100). One group received only phosphate-buffered saline (PBS; leaf control, LC).

All groups were incubated at 25 °C for 1 h, with moisture maintained by placing the 60 mm Petri plates in larger ones (90 mm) containing 10 mL of PBS. Leaves were collected at 0, 6, 12, 18, and 24 h and processed by orbital shaking (130 rpm) in 10 mL sterile PBS for 30 min at 25 °C. Phage titre was determined in triplicate using the double-agar layer method according to [30]. Bacterial concentration was determined in triplicate using the drop-plate method on TSA medium after 48 h at 25 °C. This experiment was repeated three times on different dates.

### 2.13. Phage DNA Extraction

The nucleic acid of the phage virions was extracted using the ZymoBIOMICS™ DNA Miniprep Kit (Zymo Research; Irvine, CA, USA), as outlined in the manufacturer’s instructions. The extracted DNA was then quantified with a Qubit 3.0 Fluorometer (Thermo Scientific; Waltham, MA, USA) using the Qubit 1X dsDNA assay.

### 2.14. Genomic Analysis

The initial sequence reads were checked for quality using FASTQC v0.11.9 (FastQC source: Bioinformatics Group at the Babraham Institute, Cambridge, UK). Low-quality reads and adaptors were trimmed with Trimmomatic v0.39 using the parameters ILLUMINACLIP:adaptors.fasta:2:30:1, LEADING:8, TRAILING:8, SLIDINGWINDOW:4:15, and MINLEN:100 [56]. High-quality reads were assembled de novo with SPAdes v3.13.1 using the –careful parameter [57]. Assembly graphs were visualized and inspected with Bandage v0.8.1 [58]. Reads were subsequently mapped to the assembly using BBMap v38.18 [59] to assess contig coverage, and low-coverage or spurious contigs were manually removed. The assembly was polished with Pilon v1.24 [60]. Genome termini could not be determined using PhageTerm [61]; however, analysis with apc.pl (https://github.com/jfass/apc, accessed on 20 March 2023) indicated circular permutation, and redundant sequence regions were removed. The genome was manually reordered to align with its closest related phage as described by [62]. Among the three isolated phages, PSA-2T demonstrated the most rapid and sustained bacterial inactivation, both as an individual suspension and in phage cocktails, and was therefore selected for full genomic characterization. The complete PSA-2T genome was compared against the NCBI GenBank database using BLASTN tool from BLAST+ v2.13.0 (somewhat similar sequences option) to identify closely related phages. Genome completeness and contamination were evaluated using CheckV v1.0.1 [63]. Functional genome annotation was conducted with Pharokka v1.3.2 [64] to identify coding DNA sequences (CDS), tRNAs, tmRNAs, CRISPR elements, virulence factors (VFs), toxins, and antimicrobial resistance genes (ARGs). Predicted CDS were assigned to functional categories according to the PHROGs database [65].

### 2.15. Viral Proteomic Tree

Using computational time of the SuperComputer System of the Institute for Chemical Research of Kyoto University (Kyoto, Japan), the web-based ViPTree application (https://www.genome.jp/viptree accessed on 30 January 2025) [66] was used to analyze similarities and relationships between phage PSA-2T and other prokaryotic dsDNA viruses.

### 2.16. Statistical Analyses

All statistical analyses were conducted using GraphPad Prism v6.01 (GraphPad Software, San Diego, CA, USA). Data were expressed as mean ± standard deviation (SD) from three independent experiments. The assumption of normality was verified using the Kolmogorov–Smirnov test, and homogeneity of variances was assessed by Levene’s test. Differences in bacterial and phage concentrations across treatments and time points were analyzed using two-way analysis of variance (ANOVA), followed by Bonferroni’s post hoc correction for multiple comparisons (Section 3.6, Section 3.8 and Section 3.10). The effects of environmental parameters (temperature, pH, and solar radiation) on phage viability (Section 3.7) were evaluated using one-way ANOVA with Tukey’s post hoc test for pairwise comparisons. Statistical significance was established at *p* < 0.05.

## 3. Results

### 3.1. Phage Lysis Plaques and Phage Morphology

Transmission electron microscopy (TEM) revealed that all three phages possess icosahedral heads and long, flexible tails, characteristic of the Caudoviricetes class with siphovirus morphotypes (Figure S1a–c). Phage PSA-2T exhibited a head diameter of 82 ± 1 nm and a tail length of 235 ± 4 nm, PSA-6F a head of 57 ± 2 nm and tail of 250 ± 10 nm, and PSA-7F a head of 83 ± 5 nm with a tail of 255 ± 15 nm.

### 3.2. SDS-PAGE Analysis of Phage Structural Proteins

The structural protein composition of the three phages was analyzed by SDS–PAGE (Figure S2). Distinct banding patterns were observed, indicating differences in structural protein composition. PSA-2T displayed 27 polypeptide bands (≈19–125 kDa), PSA-6F 24 bands (≈19–174 kDa), and PSA-7F 34 bands (≈19–173 kDa). Prominent bands correspond to the expected molecular masses of major capsid and tail proteins, consistent with siphovirus morphology.

### 3.3. Phage Host Range Analysis and Efficiency of Plating (EOP)

Spot and EOP assays demonstrated that all three phages lysed their primary host, *P. syringae* pv. *actinidiae* CRA-FRU 14.10 and two additional strains: *P. syringae* pv. *actinidiae* CRA-FRU 12.54 and *P. syringae* pv. *syringae* (Table 1). No lytic activity was detected against the remaining 23 strains tested.

### 3.4. Adsorption Curves

**Figure 1:**
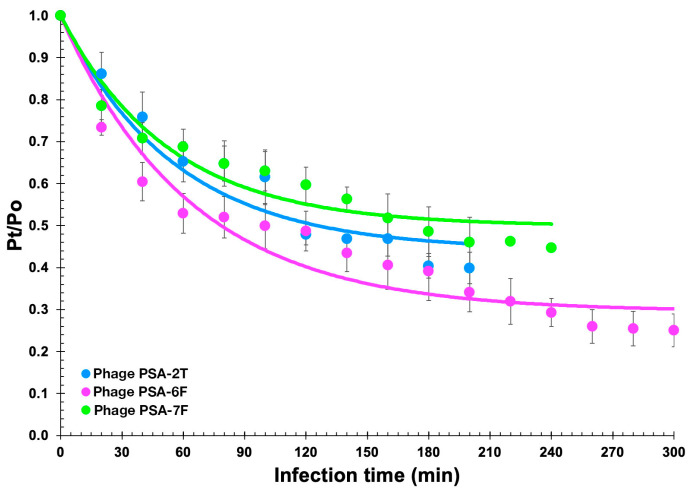
Adsorption curves of phages PSA-2T, PSA-6F and PSA-7F in the presence of *P. syringae* pv. *actinidiae* CRA-FRU 14.10 as the host. Solid lines represent the non-linear fitting of the normalized phage adsorption decay model in Section 2.6 to the experimental data. Values are the means of three independent assays. Error bars represent the standard deviations. Some of the error bars are occluded by the data points due to being very small.

Phage adsorption kinetics revealed moderately slow attachment rates across all isolates (Figure 1). Approximately 40% of PSA-2T particles adsorbed to *P. syringae* pv. *actinidiae* CRA-FRU 14.10 within 100 min, increasing to 60% by 200 min. PSA-6F exhibited 50% adsorption within 100 min and 75% by 260 min, whereas PSA-7F showed 40% and 55% adsorption at 120 and 240 min, respectively. Nonlinear regression of normalized data (Section 2.6) yielded adsorption constants of 1.00 × 10^−10^, 1.11 × 10^−10^, and 9.75 × 10^−11^ mL min^−1^ cell^−1^ for PSA-2T, PSA-6F, and PSA-7F, respectively. Corresponding desorption rates were 7.92 × 10^−3^, 4.65 × 10^−3^, and 9.35 × 10^−3^ min^−1^, with coefficients of determination of 0.903, 0.798, and 0.914, respectively.

### 3.5. One-Step Growth Curves

**Figure 2:**
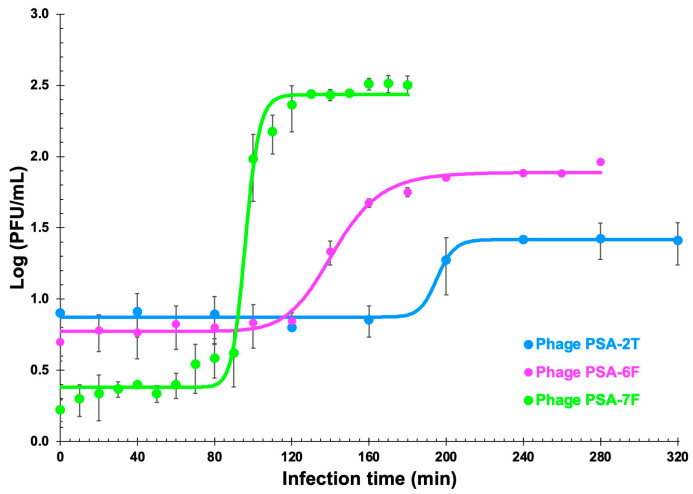
One-step growth curves of phages PSA-2T, PSA-6F and PSA-7F within cells of the host (*P. syringae* pv. *actinidiae* CRA-FRU 14.10). Solid lines represent the non-linear fitting of the four-parameter logistic regression model depicted in Section 2.7 to the experimental phage growth data. Values are the means of three independent assays. Error bars represent the standard deviations. Some of the error bars are occluded by the data points due to being very small.

One-step growth experiments revealed typical triphasic curves comprising latent, rise, and plateau phases (Figure 2). Phage PSA-2T exhibited a latent period of 230 min and a burst size of 3 PFU per host cell, PSA-6F a latent period of 200 min with a burst size of 16 PFU per host cell, and PSA-7F a latent period of 120 min and a burst size of 164 PFU per host cell. These results indicate considerable variation in replication dynamics among the three isolates.

### 3.6. Bacterial Inactivation by Single Phage Suspensions and Phage Cocktails

**Figure 3:**
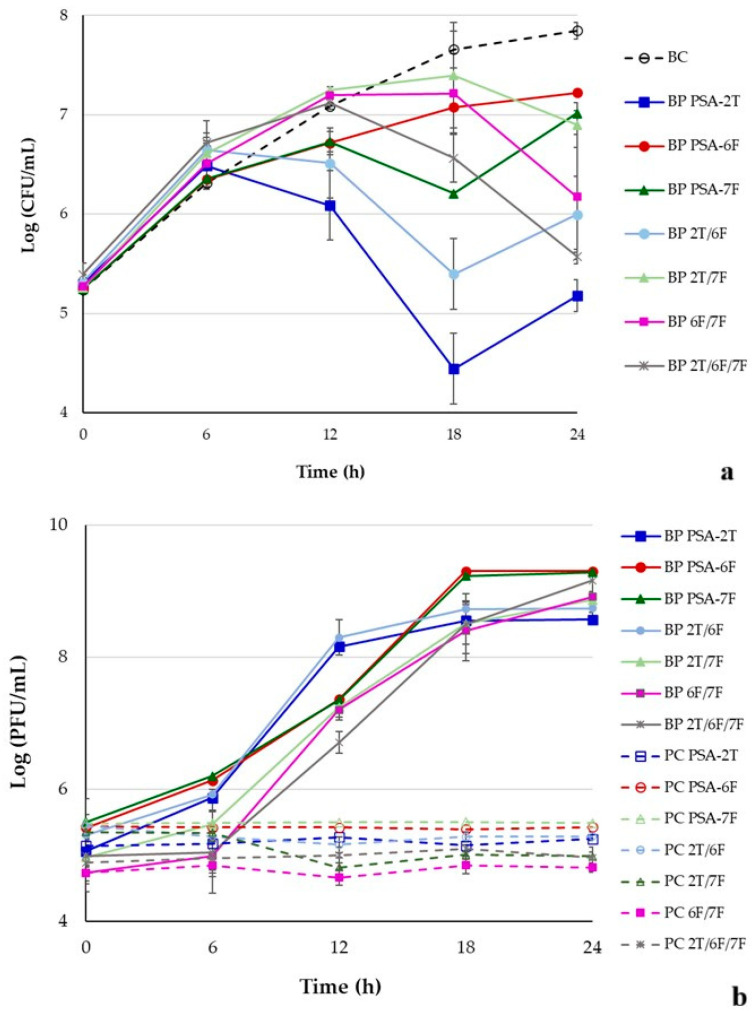
In vitro inactivation of *P. syringae* pv. *actinidiae* CRA-FRU 14.10 by the three phages (PSA-2T, PSA-6F, and PSA-7F) and phage cocktails PSA-2T/PSA-6F, PSA-2T/PSA-7F, PSA-6F/PSA-7F, and PSA-2T/PSA-6F/PSA-7F, at an MOI of 1 over a 24 h-timeframe treatment. (**a**) Bacterial concentration: BC, bacterial control; BP, bacteria plus phage; (**b**) Phage concentration: PC, phage control; BP, bacteria plus phage. Each data point is the mean of three independent experiments, and error bars depict the standard deviation. Some of the error bars are occluded by the data points due to being very small.

Bacterial density in the control (BC) increased by 2.6 log CFU/mL over 24 h (ANOVA, *p* < 0.05; Figure 3a). In contrast, phage treatments led to maximum reductions of 3.2, 0.6, and 1.5 log CFU/mL for PSA-2T, PSA-6F, and PSA-7F, respectively, after 18 h of incubation (ANOVA, *p* < 0.05, Figure 3a). During the initial 6 h, bacterial growth in all treatments was comparable to the control (ANOVA, *p* > 0.05). However, from 12 h onward, bacterial reduction by PSA-2T was significantly greater than that achieved by PSA-6F or PSA-7F (ANOVA, *p* < 0.05). PSA-6F and PSA-7F showed no significant difference until 18 h, after which PSA-7F caused a greater reduction (1.5 log CFU/mL vs. 0.6 log CFU/mL, Figure 3a).

Phage titres in control samples (PC) remained stable (ANOVA, *p* > 0.05; Figure 3b). In the presence of host cells, titres increased by 3.5, 3.9, and 3.9 log PFU/mL for PSA-2T, PSA-6F, and PSA-7F, respectively (ANOVA, *p* < 0.05, Figure 3b), confirming active phage replication.

Combined treatments produced maximum bacterial reductions of 2.2, 0.8, 1.6, and 2.2 log CFU/mL for the PSA-2T/PSA-6F, PSA-2T/PSA-7F, PSA-6F/PSA-7F, and PSA-2T/PSA-6F/PSA-7F cocktails, respectively. During the first 6 h, bacterial counts remained similar to controls, but after 12–18 h, the PSA-2T/PSA-6F cocktail achieved the most rapid inactivation (ANOVA, *p* < 0.05, Figure 3a). By 24 h, the three-phage cocktail produced the greatest overall reduction, significantly outperforming PSA-2T/PSA-7F (ANOVA, *p* < 0.05) and showing comparable efficacy to PSA-2T/PSA-6F and PSA-6F/PSA-7F.

Phage titres in control samples were stable throughout (ANOVA, *p* > 0.05; Figure 3b). When cocktails were incubated with *P. syringae* pv. *actinidiae* CRA-FRU 14.10, titres increased by 3.4, 3.9, 4.2, and 4.2 log PFU/mL for PSA-2T/PSA-6F, PSA-2T/PSA-7F, PSA-6F/PSA-7F, and PSA-2T/PSA-6F/PSA-7F, respectively (ANOVA, *p* < 0.05, Figure 3b), indicating efficient amplification under co-infection conditions.

### 3.7. Rate of Emergence of Phage-Resistant Bacterial Mutants

*Pseudomonas syringae* pv. *actinidiae* CRA-FRU 14.10 showed different rates of emergence of phage-resistant mutants for single phage suspensions and phage cocktails (Table 2). The frequency of bacterial mutants resistant to phage PSA-2T, PSA-6F, and PSA-7F was higher than that observed with phage cocktails (Table 2).

### 3.8. Evaluation of Environmental Factors on Phage Viability

**Figure 4:**
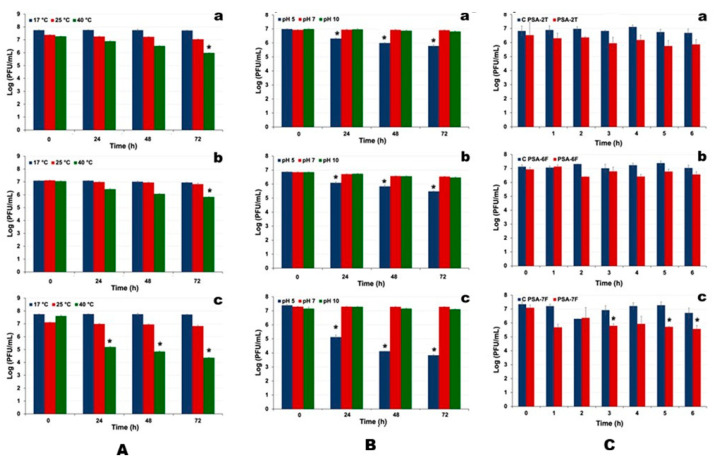
Survival of phages PSA-2T (**a**), PSA-6F (**b**), and PSA-7F (**c**) after exposure to (**A**) different temperatures (17 °C, 25 °C, and 40 °C) and (**B**) different pH values (5, 7, and 10) for 72 h, and (**C**) solar radiation for 6 h. The values represent the mean of three independent experiments, with error bars indicating standard deviation; * *p* < 0.05.

Phages PSA-2T, PSA-6F, and PSA-7F remained stable for 72 h at 17 °C and 25 °C (ANOVA, *p* > 0.05; Figure 4A(a–c)). At 40 °C, titres declined significantly (ANOVA, *p* < 0.05), with maximum reductions of 1.3, 1.2, and 3.2 log PFU/mL for PSA-2T, PSA-6F, and PSA-7F, respectively.

All phages were stable at pH 7 and 10, whereas at pH 5, significant titre decreases occurred after 72 h (ANOVA, *p* < 0.05), reaching 1.2, 1.4, and 3.6 log PFU/mL reductions for PSA-2T, PSA-6F, and PSA-7F, respectively (Figure 4B(a–c)).

Exposure to solar radiation for 6 h caused limited titre losses in PSA-2T (0.7 log PFU/mL) and PSA-6F (0.4 log PFU/mL), but a greater reduction in PSA-7F (1.5 log PFU/mL) (ANOVA, *p* < 0.05; Figure 4C(a–c)).

Overall, PSA-2T and PSA-6F demonstrated superior thermal, pH, and photostability compared with PSA-7F.

### 3.9. Bacterial Inactivation Curves with Phage PSA-2T

**Figure 5:**
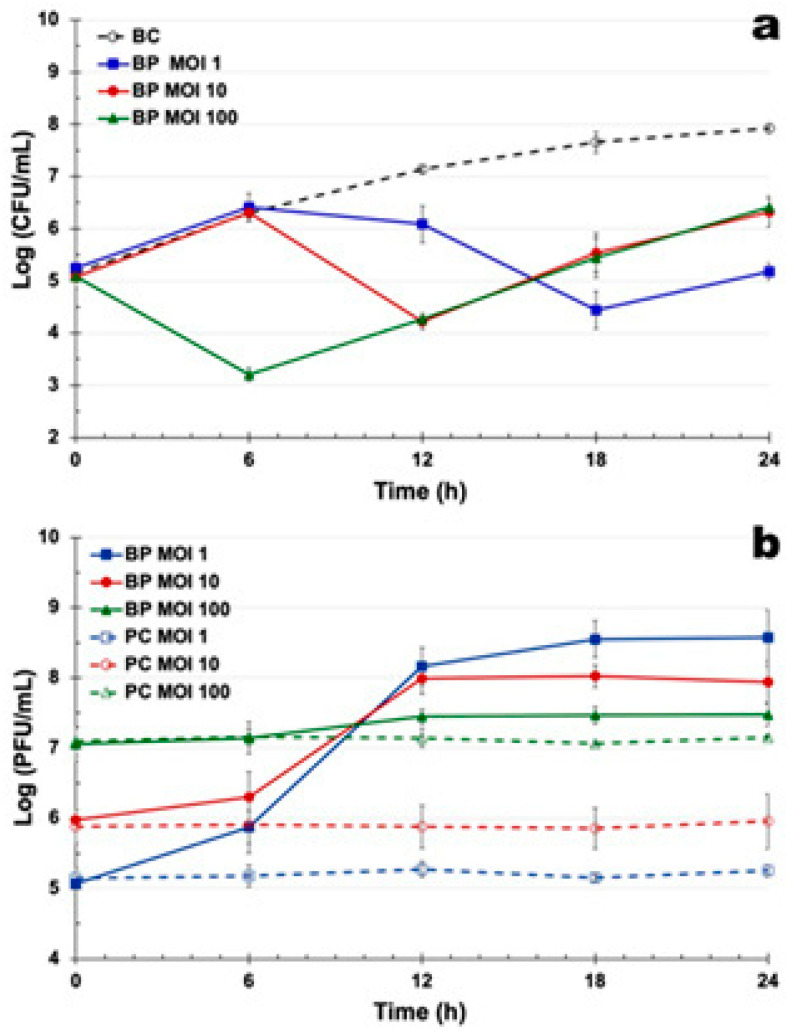
In vitro inactivation of *P. syringae* pv. *actinidiae* CRA-FRU 14.10 by phage PSA-2T alone at MOI 1, 10, and 100 over a 24 h-timeframe treatment. (**a**) Bacterial concentration: BC, bacterial control; BP, bacteria plus phage; (**b**) Phage concentration: PC, phage control; BP, bacteria plus phage. Each data point is the mean of three independent experiments, and error bars depict the standard deviation. Some of the error bars are occluded by the data points due to being very small.

In the BC, bacterial density increased by 2.8 log CFU/mL after 24 h (Figure 5a). Maximum bacterial reductions of 3.2, 2.9, and 3.1 log CFU/mL were achieved with PSA-2T at MOIs of 1, 10, and 100, respectively, after 18, 12, and 6 h of incubation relative to the BC (ANOVA, *p* < 0.05; Figure 5a). During the first 6 h, reductions at MOIs 1 and 10 were similar (ANOVA, *p* > 0.05), but at MOI 100, a faster inactivation was observed (ANOVA, *p* < 0.05). After 18–24 h, MOI 1 sustained the highest overall inhibition, whereas higher MOIs showed partial bacterial regrowth (ANOVA, *p* < 0.05).

Phage titres in controls remained constant (ANOVA, *p* > 0.05; Figure 5b). When co-incubated with the host, PSA-2T titres increased by 3.5 and 2.0 log PFU/mL at MOIs 1 and 10 (Figure 5b), respectively, after 18 h and 12 h, but did not change significantly at MOI 100, suggesting reduced replication efficiency at high phage-to-host ratios.

### 3.10. Phage Treatment on Kiwifruit Plant Leaves Artificially Contaminated with P. syringae pv. actinidiae

**Figure 6:**
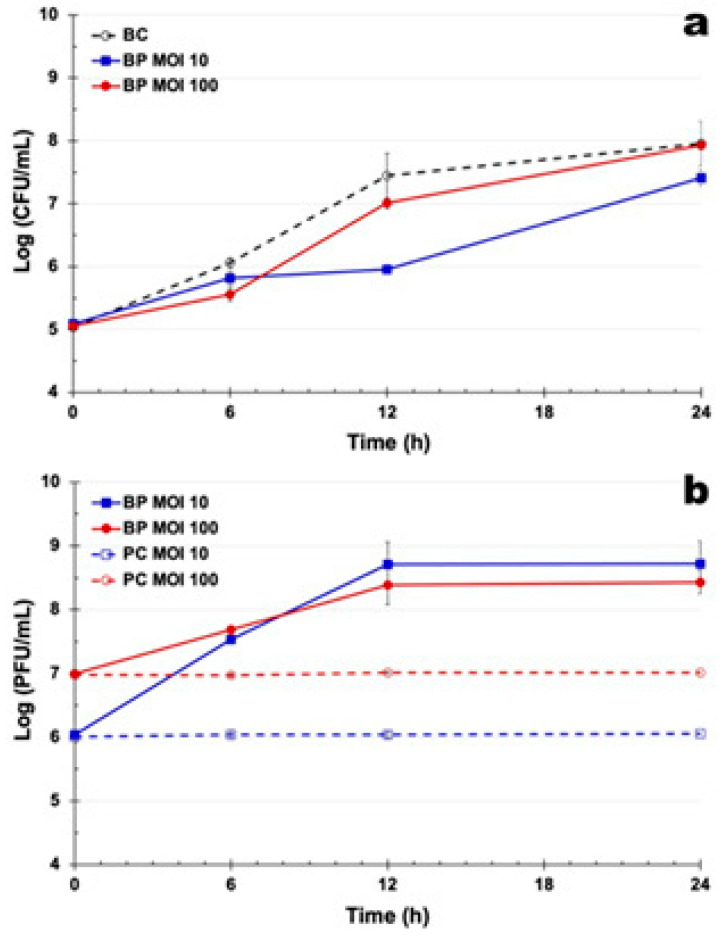
Phage treatment of kiwifruit leaves artificially inoculated with *P. syringae* pv. *actinidiae* CRA-FRU 14.10, using phage PSA-2T at multiplicities of infection (MOI) of 10 and 1000 over a 24-h period. (**a**) Bacterial concentration: BC, bacterial control; BP, bacteria plus phage; (**b**) Phage concentration: PC, phage control; BP, bacteria plus phage. Values are the mean of three independent experiments, whereas error bars represent the standard deviation. Some of the error bars are occluded by the data points due to being very small.

*Pseudomonas syringae* pv. *actinidiae* was not detected in the naturally contaminated leaves prior to inoculation (below the detection limit). In the bacterial control, population density increased by 2.9 log CFU/mL over 24 h (ANOVA, *p* < 0.05; Figure 6a). Treatment with PSA-2T resulted in a maximum reduction of 1.5 log CFU/mL at MOI 10 after 12 h (ANOVA, *p* < 0.05), while the reduction at MOI 100 (0.5 log CFU/mL) was not significantly different from the control (ANOVA, *p* > 0.05). After 24 h, bacterial counts at both MOIs were similar (ANOVA, *p* > 0.05).

Phage titres in control samples remained stable (ANOVA, *p* > 0.05; Figure 6b). In infected samples, titres increased by 2.7 and 1.4 log PFU/mL at MOIs 10 and 100 (Figure 6b), respectively, after 12 h (ANOVA, *p* < 0.05), confirming active replication on the leaf surface. These results indicate that PSA-2T can reduce *P. syringae* pv. *actinidiae* populations on kiwifruit leaves, particularly at moderate MOIs, though environmental factors may limit long-term efficacy.

### 3.11. Genome Analysis

**Figure 7:**
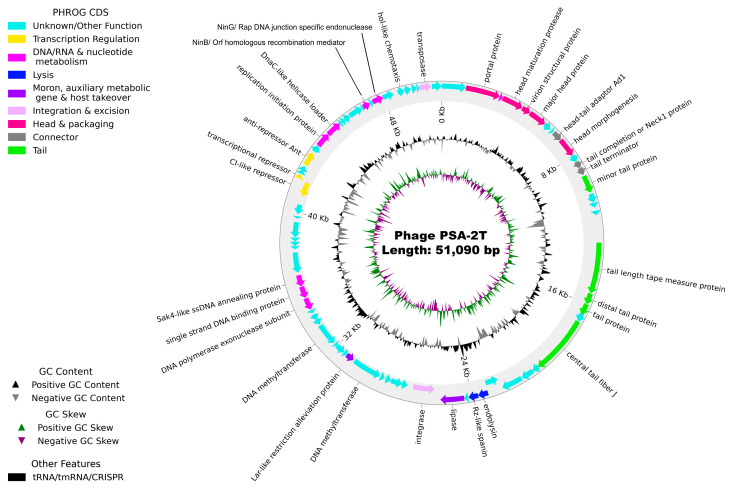
Annotated genome map of phage PSA-2T, displaying GC skew, G + C content, and predicted coding sequences (CDSs) according to the annotation in Supplementary Table S1. The outer circle with arrow-headed bands represents the CDS, color-coded according to the functional category of the predicted genes in the direction of the transcription. The innermost ring represents the genome GC skew (green/violet), followed by GC content (black/grey). The labels show the predicted functions of the functional CDSs, color-coded by PHROGs category. The arrows represent the direction of transcription (strand + or −).

The genome of phage PSA-2T is a double-stranded DNA (dsDNA) molecule of 51,090 bp, with a GC content of 58.6%, encoding 77 predicted coding sequences (CDSs). CheckV analysis indicated 100% completeness and no bacterial host contamination. The genome exhibited a coding density of 91%, of which approximately 43% of CDSs were functionally annotated, including genes related to DNA replication, structural components, packaging, and host lysis (e.g., endolysin) (Figure 7). The remaining 57% of CDSs were annotated as hypothetical proteins. No genes associated with toxins, ARGs, virulence factors, tRNA, tmRNA, CRISPR arrays, or lysogenic markers (e.g., integrase, transposase, or repressor/anti-repressor genes) were detected (Supplementary Table S1; Figure 7).

Comparative genomic analysis revealed that PSA-2T shares complete nucleotide identity and genome coverage with *Pseudomonas* phage phiPSA1 (GenBank Accession No. NC_024365), a member of the *Siphoviridae* family (Figure 7). In broader phylogenomic comparisons, PSA-2T clustered closely with phiPSA1 and *Pseudomonas* phage YMC11/02/R656, forming a distinct branch within the *Siphoviridae* lineage (Figure S3). Pairwise genome alignments indicated 100% sequence identity with phiPSA1 and 48.7% identity with YMC11/02/R656 (Figure S4).

## 4. Discussion

Developing environmentally friendly and effective biocontrol strategies to control infections by the phytopathogen *P. syringae* pv. *actinidiae* in kiwifruit plantations while minimizing the risk of bacterial resistance remains a significant challenge. In this study, three novel isolated lytic phages targeting *P. syringae* pv. *actinidiae* (PSA-2T, PSA-6F, and PSA-7F) were physicochemically and biologically characterized. Additionally, the most effective phage, PSA-2T, underwent genomic analysis. All phages and their cocktail combinations were evaluated in vitro (planktonic assays), and PSA-2T was further tested ex vivo for its ability to inactivate *P. syringae* pv. *actinidiae* CRA-FRU 14.10 on artificially contaminated kiwifruit plant leaves. This study provides the first detailed characterization of a *P. syringae* pv. *actinidiae*-specific siphovirus morphotype isolated in Aveiro, Portugal.

Although phages are typically characterized based on their isolation host, they may also infect other bacterial strains that share similar surface receptors [67]. Their host range is largely determined by specific tail fibers or spikes, which mediate the initial, reversible adsorption to susceptible bacteria and thereby control host specificity and infection efficiency [68]. Spot test and EOP assays revealed that all three phages could infect two additional *Pseudomonas* strains (Table 1, indicating a relatively narrow but overlapping host range. Comparable findings were reported by Pinheiro et al. [30], who showed that phage Φ6, besides infecting its original host (*P. syringae* pv. *syringae* DSM 21482), also infected *P. syringae* pv. *actinidiae* CRA-FRU 12.54 and *P. syringae* pv. *actinidiae* CRA-FRU 14.10. The similar host ranges observed among PSA-2T, PSA-6F, and PSA-7F may reflect similarities in their tail fiber structures, as inferred from their morphological profiles. These observations reinforce the need to isolate additional lytic phages against *P. syringae* pv. *actinidiae* to assemble broad-spectrum phage cocktails capable of controlling a wider diversity of strains.

Phage infection begins with recognition and adsorption to a host cell receptor. The adsorption rates of PSA-2T and PSA-6F onto *P. syringae* pv. *actinidiae* CRA-FRU 14.10 were consistent with those reported for other *Pseudomonas* phage–host systems, including phages Φ6 [30,69] and phages ph0031 and ph0034 [70]. Phage PSA-7F exhibited a slightly lower rate but remained within the range observed for phage Φ6 with *P. syringae* pv. *actinidiae* CRA-FRU 12.54 [30]. Interestingly, an inverse relationship was observed between adsorption rate and the time to maximum bacterial inactivation, challenging the assumption that faster adsorption necessarily leads to more efficient biocontrol. Although PSA-7F displayed a higher adsorption rate than PSA-2T, the latter achieved greater in vitro bacterial inactivation relative to the control. A plausible explanation, consistent with spatial infection models, is that slower-adsorbing phages may diffuse more uniformly before attachment, promoting synchronized infections across the bacterial population. In contrast, phages with rapid adsorption rates may bind quickly to nearby cells, causing localized infections and early depletion of susceptible hosts, which can limit further spread [71,72]. This observation underscores that the adsorption rate does not always positively correlate with biocontrol efficiency, particularly in spatially structured environments [73]. Balancing adsorption kinetics with dispersal ability may therefore be more critical for optimizing *P. syringae* pv. *actinidiae* biocontrol. Such insights could inform more predictive frameworks for selecting agricultural biocontrol candidates [74,75] and for adjusting phage concentrations to maximize infection efficiency [53].

Phage replication kinetics revealed distinct growth characteristics for each isolate. Phage PSA-7F displayed the shortest latency period (120 min) and the highest burst size (164 virions cell^−1^). In contrast, phages PSA-2T and PSA-6F exhibited longer latency periods (230 min and 200 min, respectively) and smaller burst sizes (3 and 6 virions cell^−1^). Several studies suggest that high virion yields and short latent periods generally enhance phage-based biocontrol efficiency [50,76,77,78,79]. However, larger burst sizes are often associated with extended latency periods [72], and this pattern was not observed in the present study. Both PSA-2T and PSA-6F showed unusually low burst sizes combined with prolonged latency, a combination that may reduce replication efficiency but could also influence infection dynamics in structured environments.

In vitro inactivation of planktonic *P. syringae* pv. *actinidiae* by phage PSA-2T was much more effective than the other two phages at an MOI of 1. Despite this efficacy, mild resistance to PSA-2T emerged after 18 h, although bacterial counts remained substantially lower than in the untreated control. Similar results were reported by [30], where phage Φ6 achieved maximum reductions of 2.2 and 1.9 log CFU/mL against *P. syringae* pv. *actinidiae* CRA-FRU 12.54 and 14.10, respectively. The three phages showed relatively slow inactivation kinetics, reaching maximum reductions only after 18 h, likely due to their low adsorption rates and long latent periods.

While the emergence of phage-resistant bacteria is a well-known limitation in biocontrol applications [80,81], several studies indicate that this can be mitigated by formulating cocktails comprising lytic phages with distinct adsorption and replication mechanisms [30,79,81,82,83,84]. At MOI 1, the PSA-2T/PSA-6F cocktail achieved significantly greater bacterial inactivation after 12 and 18 h compared with the other two-phage mixtures and with the tripartite cocktail. However, by the end of the treatment period, the three-phage combination was the most effective overall, producing the largest reduction in bacterial load with no detectable resistance development. The comparable performance of the PSA-2T/PSA-6F and PSA-6F/PSA-7F cocktails likely reflects overlapping receptor specificities, as PSA-2T appears to target the same bacterial receptor as the other two phages. Mild regrowth observed in the PSA-2T/PSA-6F treatment supports this interpretation. Collectively, these results indicate that PSA-2T was the main contributor to bacterial reduction, either alone or within phage mixtures. They also emphasize the importance of rational cocktail design—combining phages with complementary receptor specificities and infection kinetics—to enhance efficacy and minimize resistance development [25]. Further genomic and receptor-binding studies are warranted to elucidate these interactions and to optimize phage combinations for environmental or clinical use.

The emergence of phage-resistant *P. syringae* pv. *actinidiae* strains remain a major challenge for sustainable phage biocontrol in kiwifruit cultivation. The frequency of spontaneous phage-resistant mutants was of similar magnitude across individual phages and cocktails (Table 2). Yet, these values were 1–2 orders of magnitude higher than those reported for *P. coronafaciens* pv. *garcae* with phages ph002F and ph004F [85] and for *P. aeruginosa* PAO1 with phage PA5P2 [86]. Resistance frequencies were also 3–5 orders of magnitude higher than those reported for other *Pseudomonas* phytopathogens treated with phages such as KIL3, KIL4 [87], PaP1 [88], LUZ19v, and PAK_P1 [89]. Although phages represent a highly specific and environmentally benign alternative to chemical bactericides [28,90], their long-term effectiveness can be compromised by rapid bacterial resistance [91,92]. Bacterial cells acquire resistance to the phage after 3–5 days following contact with the phage virions, and therefore, new infection cycles begin. To counter this, cocktails targeting distinct bacterial receptors can substantially delay resistance emergence [82,84]. Moreover, integrated disease management strategies that combine phage biocontrol with complementary approaches—such as the use of resistant plant cultivars, antimicrobial peptides, or plant-derived compounds—are increasingly recognized as essential for durable protection. To date, only one study has explored combination therapies against *P. syringae* pv. *actinidiae*, testing phages in conjunction with carvacrol [42]. Synergistic integration of phages with other control measures may reduce selective pressure and expand the protective spectrum [93,94]. Using agents with distinct mechanisms of action decreases the likelihood of bacterial adaptation to all selective pressures simultaneously [95,96]. This integrated approach aligns with sustainable agriculture and integrated pest management principles, promoting resilient protection strategies against evolving microbial threats.

The three tested phages demonstrated good thermal and pH stability under conditions relevant to field application. PSA-6F was more thermally tolerant at 40 °C than PSA-2T and PSA-7F, which experienced greater loss of infectivity; however, such elevated temperatures are uncommon in kiwifruit orchards, limiting practical concern. All phages remained stable at neutral and alkaline pH (7.0 and 10.0), whereas acidic conditions (pH 5.0) substantially reduced activity. Given that kiwifruit leaf surface pH is typically near neutral, these results support the potential in planta use of these phages. Regarding solar exposure, PSA-2T and PSA-6F retained infectivity with minimal losses, while PSA-7F was more sensitive to UV radiation. These findings align with prior reports and underscore the importance of environmental conditions in determining phage efficacy in field applications. Phage preparations can be formulated to enhance resistance to ultraviolet (UV) radiation. UV exposure can directly affect viruses by modifying their genetic material (DNA or RNA) or by damaging viral proteins and lipids; however, inactivation is primarily driven by nucleic acid damage [55,97]. Absorbed UV radiation induces photochemical reactions that cause the fusion of adjacent pyrimidines into covalently linked dimers—uracil/cytosine dimers in RNA and thymine/cytosine dimers in DNA [55,97]. Additional mechanisms of UV-mediated viral inactivation include RNA–protein cross-linking [98] and site-specific damage to RNA or proteins through energy transfer between these molecules [99]. Collectively, these effects impair the ability of infected cells to replicate, thereby compromising phage survival [55,97]. However, this limitation can be mitigated by incorporating phages into micro- and/or nanocarriers, applying them at high titres, and timing their deployment for late in the day or during nighttime, when UV radiation is minimal [100,101,102,103].

Phage PSA-2T demonstrated the most rapid and sustained bacterial inactivation, both as a standalone treatment and within phage cocktails, underscoring its strong potential for future field applications. Accordingly, PSA-2T was selected for further detailed characterization. Previous studies have shown that increasing MOI generally accelerates and enhances bacterial reduction [50,70,77,104,105,106]. Consistently, in this work, increasing the MOI significantly improved bacterial reduction within the first 6 h. However, this was followed by a marked rise in phage-resistant populations, particularly at MOI 100 (after 6 h of incubation) and MOI 10 (after 12 h of incubation). At MOI 1, resistance developed more slowly, only becoming evident after 18 h of incubation. Interestingly, by the treatment’s end, MOI 1 resulted in a more sustained bacterial reduction than higher MOIs, aligning with the resistance emergence data (Table 2).

In phage treatment experiments on kiwifruit leaves artificially contaminated with bacteria, the phage PSA-2T successfully infected and killed *P. syringae* pv. *actinidiae*. Although the final bacterial counts at MOIs 10 and 100 were not statistically significant, MOI 10 was more efficient in lowering the bacterial load during the first 12 h of treatment. The bacterial decrease was, however, less pronounced than in vitro. In suspension cultures, interactions between phage particles and bacterial cells occur more readily through random collisions. However, on leaf surfaces, the presence of a waxy cuticle restricts the mobility of both phages and bacteria, limiting their encounters and reducing net phage progeny. This likely explains the lower amplification of phage virions observed in kiwifruit leaves. To counteract this limitation, multiple rounds of phage application [107]—already common practice in phage-based biocontrol—can be implemented [4,96]. Field application could follow protocols established for copper-based compounds, spraying phages in free form or encapsulated within micro- or nanocarriers. Given phage PSA-2T’s sensitivity to abiotic factors such as temperature, applications should preferably be made at dawn or during the night period. Translating this strategy to field conditions will require further studies, beginning with whole plants under controlled laboratory conditions and progressing to naturally contaminated orchards.

The genome of phage PSA-2T was screened for lysogeny-associated genes [108], and no genes encoding known lysogenic markers—such as integrase, transposase, and repressor/anti-repressor proteins—were found. These results suggest that phage PSA-2T likely follows a strictly lytic lifestyle [67,109]. However, as over half of the predicted protein-coding genes have unknown functions, it remains possible—though unconfirmed—that some may encode known toxins, antimicrobial-resistance genes, virulent factors of bacterial origin, tRNA, tmRNA, and CRISPR sequences. Strictly lytic phages are recommended for biocontrol applications as they minimize the risk of transferring antibiotic resistance or virulence genes [110,111]. Phage PSA-2T showed strong genomic similarity with *Pseudomonas* phage phiPSA1 (100% coverage and identity; GenBank Accession No. NC_024365); both share a siphovirus morphotype and are highly specific to *P. syringae* pv. *actinidiae,* showing no infectivity toward other *Pseudomonas* species.

The demonstrated ability of phage PSA-2T to reduce *P. syringae* pv. *actinidiae* CRA-FRU 14.10 concentrations both in vitro and on kiwifruit leaves represent a promising step toward developing an effective alternative to conventional copper- and antibiotic-based treatments for kiwifruit bacterial canker. Nonetheless, translating this strategy to field applications will require in planta validation using both kiwifruit plant seedlings and mature plants, together with a phage cocktail integrating different lytic phages capable of infecting a broader spectrum of virulent *P. syringae* pv. *actinidiae* strains.

## 5. Conclusions

The results of this study strongly support the use of phages for controlling infections caused by *P. syringae* pv. *actinidiae* in kiwifruit plants. In particular, phage PSA-2T demonstrates significant potential as an effective alternative to conventional chemical control strategies currently employed in orchards. To enhance efficacy and mitigate the emergence of phage-resistant strains, the isolation, characterization, and selection of additional lytic phages for inclusion in broad-host-range phage cocktails will be essential. Moreover, repeated treatment cycles are likely to improve inactivation efficiency while further reducing the risk of bacterial regrowth. The observed in vitro and ex vivo suppression of *P. syringae* pv. *actinidiae* by phage PSA-2T represents an important step toward the development of sustainable and eco-friendly strategies for managing bacterial canker in kiwifruit.

## Figures and Tables

**Table 1:** The host range of phages PSA-2T, PSA-6F, and PSA-7F was determined for 26 bacterial strains. Clear lysis zone (+) and no lysis zone (−). An EOP value of 100% was considered for the host strain.

Strains	Phages					
	PSA-2T		PSA-6F		PSA-7F	
	Spot Test	EOP (%)	Spot Test	EOP (%)	Spot Test	EOP (%)
*P. syringae* pv. *actinidiae* CRA-FRU 14.10 (host)	+	100	+	100	+	100
*P. syringae* pv. *actinidiae* CRA-FRU 12.54	+	127 ± 4	+	99 ± 16	+	159 ± 13
*P. syringae* pv. *actinidiae* CRA-FRU 8.43	−	0	−	0	−	0
*Pseudomonas syringae* pv. *syringae* DSM 21482	+	120 ± 4	+	90 ± 6	+	148 ± 11
*Pseudomonas* sp. *X047434*	−	0	−	0	−	0
*Pseudomonas* sp. EF627998	−	0	−	0	−	0
*Pseudomonas* sp. EF628000	−	0	−	0	−	0
*Pseudomonas* sp. AF411853	−	0	−	0	−	0
*Pseudomonas* sp. HF679142	−	0	−	0	−	0
*Pseudomonas* sp. AB772943	−	0	−	0	−	0
*Pseudomonas* sp. EU306338	−	0	−	0	−	0
*Pseudomonas* sp. GU966669	−	0	−	0	−	0
*Pseudomonas* sp. AY332207	−	0	−	0	−	0
*Pseudomonas* sp. JN033360	−	0	−	0	−	0
*Pseudomonas putida* JQ619028	−	0	−	0	−	0
*Pseudomonas putida* JQ824856	−	0	−	0	−	0
*Pseudomonas stutzeri* EU167940	−	0	−	0	−	0
*Pseudomonas rhodesiae* JX994152	−	0	−	0	−	0
*Pseudomonas aeruginosa* ATCC 27853	−	0	−	0	−	0
*Aeromonas hydrophila* ATCC 7966	−	0	−	0	−	0
*Escherichia coli* ATCC 13706	−	0	−	0	−	0
*Escherichia coli* ATCC 25922	−	0	−	0	−	0
*Staphylococcus aureus* ATCC 6538	−	0	−	0	−	0
*Salmonella* Typhimurium ATCC 14028	−	0	−	0	−	0
*Salmonella* Typhimurium ATCC 13311	−	0	−	0	−	0
*Vibrio parahaemolyticus* DSM 27657	−	0	−	0	−	0

**Table 2:** Frequency of emergence of *P. syringae* pv. *actinidiae* CRA-FRU 14.10 spontaneous bacterial mutants resistant to the phages.

Single Phages/ Phage Cocktails	Control Sample (CFU/mL)	Sample Treated with Phages (CFU/mL)	Frequency of Phage-Resistant Bacterial Mutants
PSA-2T	1.60 ± 0.56 × 10^8^	2.90 ± 0.86 × 10^6^	1.86 × 10^−2^
PSA-6F	1.41 ± 0.22 × 10^8^	9.29 ± 0.49 × 10^6^	6.58 × 10^−2^
PSA-7F	1.36 ± 0.20 × 10^8^	2.13 ± 0.15 × 10^6^	1.77 × 10^−2^
PSA-2T/PSA-6F	1.44 ± 0.24 × 10^8^	2.43 ± 0.11 × 10^6^	1.69 × 10^−2^
PSA-2T/PSA-7F	1.42 ± 0.21 × 10^8^	2.23 ± 0.49 × 10^6^	1.58 × 10^−2^
PSA-6F/PSA-7F	1.39 ± 0.25 × 10^8^	2.03 ± 0.11 × 10^6^	1.47 × 10^−2^
PSA-2T/PSA-6F/PSA-7F	1.49 ± 0.14 × 10^8^	1.51 ± 0.70 × 10^6^	1.01 × 10^−2^

## Data Availability

Data are contained within the article and Supplementary Materials.

## Supplementary Materials

The following supporting information can be downloaded at: https://www.mdpi.com/article/10.3390/pathogens14121247/s1, Table S1: Coding sequences identified in PSA-2T phage. Figure S1: Transmission electron photomicrographs of isolated PSA phages. (a: phage PSA-2T (×250k magnification); b: phage PSA-6F (×200k magnification); c: phage PSA-7F (×150k magnification)). Figure S2: Coomassie-stained electrophoretogram of the structural proteins of phages PSA-2T, PSA-6F, and PSA-7F and wide-range molecular weight markers (lanes M). Figure S3: Viral proteomic tree from ViPTree analysis of phage PSA-2T and related phages. Figure S4: Genome sequence alignment and comparison of phage PSA-2T with two other phage genomes exhibiting co-linearity detected by TBLASTX tool from BLAST+ v2.13.0 using the ViPTree server [66].

## References

[1] Food and Agriculture Organization (FAO) (2022). FAOSTAT: Crops and Livestock Products.

[2] Cameron A., Sarojini V. (2014). *Pseudomonas syringae* pv. *actinidiae*: Chemical control, resistance mechanisms and possible alternatives. Plant Pathol..

[3] Kim G.H., Kim K.H., Son K.I., Choi E.D., Lee Y.S., Jung J.S., Koh Y.J. (2016). Outbreak and spread of bacterial canker of kiwifruit caused by *Pseudomonas syringae* pv. *actinidiae* biovar 3 in Korea. Plant Pathol. J..

[4] Pereira C., Costa P., Pinheiro L., Balcão V.M., Almeida A. (2021). Kiwifruit bacterial canker: An integrative view focused on biocontrol strategies. Planta.

[5] Chapman J.R., Taylor R.K., Weir B.S., Romberg M.K., Vanneste J.L., Luck J., Alexander B.J.R. (2012). Phylogenetic relationships among global populations of *Pseudomonas syringae* pv. *actinidiae*. Phytopathology.

[6] Fujikawa T., Sawada H. (2019). Genome analysis of *Pseudomonas syringae* pv. *actinidiae* biovar 6, which produces the phytotoxins, phaseolotoxin and coronatine. Sci. Rep..

[7] Bender C.L., Alarcon-Chaidez F., Gross D.C. (1999). *Pseudomonas syringae* phytotoxins: Mode of action, regulation, and biosynthesis by peptide and polyketide synthetases. Microbiol. Mol. Biol. Rev..

[8] Fujikawa T., Sawada H. (2016). Genome analysis of the kiwifruit canker pathogen *Pseudomonas syringae* pv. *actinidiae* biovar 5. Sci. Rep..

[9] Falcone G., Campa M. (1988). Diseases Caused by Pseudomonas. Laboratory Diagnosis of Infectious Diseases.

[10] Deng S., Chang W., Que Y., Liu J., Wang H. (2023). Survival of *Pseudomonas syringae* pv. *actinidiae* in detached kiwifruit leaves at different environmental conditions. PeerJ.

[11] Balestra G.M., Mazzaglia A., Quattrucci A., Renzi M., Rossetti A. (2009). Current status of bacterial canker spread on kiwifruit in Italy. Australas. Plant Dis. Notes.

[12] Takikawa Y., Serizawa S., Ichikawa T., Tsuyumu S., Goto M. (1989). *Pseudomonas syringae* pv. *actinidae* pv. nov.: The causal bacterium of canker of kiwifruit in Japan. Jpn. J. Phytopathol..

[13] European Commission (2012). Commission implementing decision of 5 December 2012 as regards measures to prevent the introduction into and the spread within the Union of *Pseudomonas syringae* pv. *actinidiae* Takikawa, Serizawa, Ichikawa, Tsuyumu & Goto. Off. J. Eur. Union.

[14] Vogelaar M., Schenk M., Delbianco A., Graziosi I., Vos S. (2020). Pest survey card on *Pseudomonas syringae* pv. *actinidiae*. EFSA Support. Publ..

[15] Donati I., Buriani G., Cellini A., Mauri S., Costa G., Spinelli F. (2014). New insights on the bacterial canker of kiwifruit (*Pseudomonas syringae* pv. *actinidiae*). J. Berry Res..

[16] La Torre A., Iovino V., Caradonia F. (2018). Copper in plant protection: Current situation and prospects. Phytopathol. Mediterr..

[17] Vanneste J., Kay C., Onorato R., Yu J., Cornish D., Spinelli F., Max S. (2011). Recent advances in the characterisation and control of *Pseudomonas syringae* pv. *actinidiae*, the causal agent of bacterial canker on kiwifruit. Acta Hortic..

[18] Young J.M. (2012). *Pseudomonas syringae* pv. *actinidiae* in New Zealand. J. Plant Pathol..

[19] Lamichhane J.R., Osdaghi E., Behlau F., Köhl J., Jones J.B., Aubertot J.-N. (2018). Thirteen decades of antimicrobial copper compounds applied in agriculture. A review. Agron. Sustain. Dev..

[20] Lee Y.S., Kim G.H., Song Y.-R., Oh C.-S., Koh Y.J., Jung J.S. (2020). Streptomycin Resistant Isolates of *Pseudomonas syringae* pv. *actinidiae* in Korea. Res. Plant Dis..

[21] Ren G., Ding Z., Pan X., Wei G., Wang P. (2022). Evaluation of the Abilities of Three Kinds of Copper-Based Nanoparticles to Control Kiwifruit Bacterial Canker. Antibiotics.

[22] Santos M.G., Nunes da Silva M., Vasconcelos M.W., Carvalho S.M.P. (2024). Scientific and technological advances in the development of sustainable disease management tools: A case study on kiwifruit bacterial canker. Front. Plant Sci..

[23] Luo J., Dai D., Lv L., Ahmed T., Chen L., Wang Y., An Q., Sun G., Li B. (2022). Advancements in the Use of Bacteriophages to Combat the Kiwifruit Canker Phytopathogen *Pseudomonas syringae* pv. *actinidiae*. Viruses.

[24] Pereira C., Costa P., Duarte J., Balcão V.M., Almeida A. (2021). Phage therapy as a potential approach in the biocontrol of pathogenic bacteria associated with shellfish consumption. Int. J. Food Microbiol..

[25] Costa P., Pereira C., Romalde J.L., Almeida A. (2024). A game of resistance: War between bacteria and phages and how phage cocktails can be the solution. Virology.

[26] Harada L.K., Pereira J.F.B., Campos W.F., Silva E.C., Moutinho C.G., Vila M.M.D.C., Oliveira J.M., Teixeira J.A., Balcão V.M., Tubino M. (2018). Insights into protein-ionic liquid interactions aiming at macromolecule delivery systems. J. Braz. Chem. Soc..

[27] Silva E.C., Quinde C.A., Cieza B., Basu A., Vila M.M.D.C., Balcão V.M. (2024). Molecular characterization and genome mechanical features of two newly isolated polyvalent bacteriophages infecting *Pseudomonas syringae* pv. *garcae*. Genes.

[28] Jones J.B., Vallad G.E., Iriarte F.B., Obradović A., Wernsing M.H., Jackson L.E., Balogh B., Hong J.C., Momol M.T. (2012). Considerations for using bacteriophages for plant disease control. Bacteriophage.

[29] Mota L.C., Silva E.C., Quinde C.A., Cieza B., Basu A., Rodrigues L.M.R., Vila M.M.D.C., Balcão V.M. (2025). Potential of a newly isolated lytic bacteriophage to control *Pseudomonas coronafaciens* pv. *garcae* in coffee plants: Molecular characterization with in vitro and ex vivo experiments. Enzym. Microb. Technol..

[30] Pinheiro L.A.M., Pereira C., Barreal M.E., Gallego P.P., Balcão V.M., Almeida A. (2020). Use of phage ϕ6 to inactivate *Pseudomonas syringae* pv. *actinidiae* in kiwifruit plants: In vitro and ex vivo experiments. Appl. Microbiol. Biotechnol..

[31] Silva E.C., Rodrigues L.M.R., Destefano S.A.L., Guerreiro Filho O., Braghini M.T., Baldo D.A., Oliveira J.M., Vila M.M.D.C., Balcão V.M. (2024). Control of coffee canker associated with *Pseudomonas coronafaciens* pv. *garcae* using a cocktail integrating two virulent polyvalent bacteriophages encapsulated in nanoparticles: In planta studies. J. Appl. Microbiol..

[32] Gimranov E., Oliveira H., Santos C., Moura L., Azeredo J. (2025). Lytic properties and genomic analysis of bacteriophage Brt_Psa3, targeting *Pseudomonas syringae* pv. *actinidiae*. Appl. Microbiol. Biotechnol..

[33] Iriarte F.B., Obradović A., Wernsing M.H., Jackson L.E., Balogh B., Hong J.A., Momol M.T., Jones J.B., Vallad G.E. (2012). Soil-based systemic delivery and phyllosphere in vivo propagation of bacteriophages: Two possible strategies for improving bacteriophage persistence for plant disease control. Bacteriophage.

[34] Abedon S.T., Herschler T.D., Stopar D. (2001). Bacteriophage Latent-Period Evolution as a Response to Resource Availability. Appl. Environ. Microbiol..

[35] Flores O., Retamales J., Núñez M., León M., Salinas P., Besoain X., Yañez C., Bastías R. (2020). Characterization of bacteriophages against *Pseudomonas syringae* pv. *actinidiae* with potential use as natural antimicrobials in kiwifruit plants. Microorganisms.

[36] Fiorillo A., Frezza D., Di Lallo G., Visconti S. (2023). A Phage Therapy Model for the Prevention of *Pseudomonas syringae* pv. *actinidiae* Infection of Kiwifruit Plants. Plant Dis..

[37] Di Lallo G., Evangelisti M., Mancuso F., Ferrante P., Marcelletti S., Tinari A., Superti F., Migliore L., D’Addabbo P., Frezza D. (2014). Isolation and partial characterization of bacteriophages infecting *Pseudomonas syringae* pv. *actinidiae*, causal agent of kiwifruit bacterial canker. J. Basic Microbiol..

[38] Frampton R.A., Acedo E.L., Young V.L., Chen D., Tong B., Taylor C., Easingwood R.A., Pitman A.R., Kleffmann T., Bostina M. (2015). Genome, proteome and structure of a T7-like bacteriophage of the kiwifruit canker phytopathogen *Pseudomonas syringae* pv. *actinidiae*. Viruses.

[39] Yu J.-G., Lim J.-A., Song Y.-R., Heu S., Kim G.H., Koh Y.J., Oh C.-S. (2016). Isolation and Characterization of Bacteriophages Against *Pseudomonas syringae* pv. *actinidiae* Causing Bacterial Canker Disease in Kiwifruit. J. Microbiol. Biotechnol..

[40] Park J., Lim J.-A., Yu J.-G., Oh C.-S. (2018). Genomic features and lytic activity of the bacteriophage PPPL-1 effective against *Pseudomonas syringae* pv. *actinidiae*, a cause of bacterial canker in kiwifruit. J. Microbiol. Biotechnol..

[41] Yin Y., Ni P., Deng B., Wang S., Xu W., Wang D. (2019). Isolation and characterisation of phages against *Pseudomonas syringae* pv. *actinidiae*. Acta Agric. Scand. Sect. B Soil Plant Sci..

[42] Ni P., Wang L., Deng B., Jiu S., Ma C., Zhang C., Almeida A., Wang D., Xu W., Wang S. (2020). Combined application of bacteriophages and carvacrol in the control of *Pseudomonas syringae* pv. *actinidiae* planktonic and biofilm forms. Microorganisms.

[43] Song Y.R., Vu N.T., Park J., Hwang I.S., Jeong H.J., Cho Y.S., Oh C.S. (2021). Phage pppl-1, a new biological agent to control bacterial canker caused by *Pseudomonas syringae* pv. *actinidiae* in kiwifruit. Antibiotics.

[44] Bai J., Liu Y., Liu M., Luo S., Cheng Y., Li G., Liu C., Wen S., Xia M., He X. (2022). Application of phage therapy against red-fleshed kiwifruit canker. Biol. Control.

[45] Firrao G., Torelli E., Polano C., Ferrante P., Ferrini F., Martini M., Marcelletti S., Scortichini M., Ermacora P. (2018). Genomic structural variations affecting virulence during clonal expansion of *Pseudomonas syringae* pv. *actinidiae* biovar 3 in Europe. Front. Microbiol..

[46] Ferrante P., Scortichini M. (2014). Frost promotes the pathogenicity of *Pseudomonas syringae* pv. *actinidiae* in *Actinidia chinensis* and *A. deliciosa* plants. Plant Pathol..

[47] Louvado A., Coelho F.J.R.C., Domingues P., Santos A.L., Gomes N.C.M., Almeida A., Cunha Â. (2012). Isolation of surfactant-resistant Pseudomonads from the estuarine surface microlayer. J. Microbiol. Biotechnol.

[48] Oliveira V., Gomes N.C.M., Almeida A., Silva A.M.S., Simões M.M.Q., Smalla K., Cunha Â. (2014). Hydrocarbon contamination and plant species determine the phylogenetic and functional diversity of endophytic degrading bacteria. Mol. Ecol..

[49] Adams M. (1959). Bacteriophages.

[50] Balcão V.M., Moreli F.C., Silva E.C., Belline B.G., Martins L.F., Rossi F.P.N., Pereira C., Vila M.M.D.C., da Silva A.M. (2022). Isolation and Molecular Characterization of a Novel Lytic Bacteriophage That Inactivates MDR *Klebsiella pneumoniae* Strains. Pharmaceutics.

[51] Kutter E., Clokie M.R., Kropinski A.M. (2009). Phage host range and efficiency of plating. Bacteriophage: Methods and Protocols.

[52] Stuer-Lauridsen B., Janzen T., Schnabl J., Johansen E. (2003). Identification of the host determinant of two prolate-headed phages infecting *Lactococcus lactis*. Virology.

[53] Hyman P., Abedon S.T. (2009). Practical methods for determining phage growth parameters. Bacteriophages: Methods and Protocols, Volume 1: Isolation, Characterization, and Interactions.

[54] Filippov A., Sergueev K.V., He Y., Huang X.Z., Gnade B.T., Mueller A.J., Fernandez-Prada C., Nikolich M.P. (2011). Bacteriophage-resistant mutants in *Yersinia pestis*: Identification of phage receptors and attenuation for mice. PLoS ONE.

[55] Bartolomeu M., Braz M., Costa P., Duarte J., Pereira C., Almeida A. (2022). Evaluation of UV-C Radiation Efficiency in the Decontamination of Inanimate Surfaces and Personal Protective Equipment Contaminated with Phage φ6. Microorganisms.

[56] Bolger A.M., Lohse M., Usadel B. (2014). Trimmomatic: A flexible trimmer for Illumina sequence data. Bioinformatics.

[57] Bankevich A., Nurk S., Antipov D., Gurevich A.A., Dvorkin M., Kulikov A.S., Lesin V.M., Nikolenko S.I., Pham S., Prjibelski A.D. (2012). SPAdes: A new genome assembly algorithm and its applications to single-cell sequencing. J. Comput. Biol..

[58] Wick R.R., Schultz M.B., Zobel J., Holt K.E. (2015). Bandage: Interactive visualization of de novo genome assemblies. Bioinformatics.

[59] Bushnell B., Rood J., Singer E. (2017). BBMerge—Accurate paired shotgun read merging via overlap. PLoS ONE.

[60] Walker B.J., Abeel T., Shea T., Priest M., Abouelliel A., Sakthikumar S., Cuomo C.A., Zeng Q., Wortman J., Young S.K. (2014). Pilon: An integrated tool for comprehensive microbial variant detection and genome assembly improvement. PLoS ONE.

[61] Garneau J.R., Depardieu F., Fortier L.C., Bikard D., Monot M. (2017). PhageTerm: A tool for fast and accurate determination of phage termini and packaging mechanism using next-generation sequencing data. Sci. Rep..

[62] Shen A., Millard A. (2021). Phage genome annotation: Where to begin and end. Phage.

[63] Nayfach S., Camargo A.P., Schulz F., Eloe-Fadrosh E., Roux S., Kyrpides N.C. (2021). CheckV assesses the quality and completeness of metagenome-assembled viral genomes. Nat. Biotechnol..

[64] Bouras G., Nepal R., Houtak G., Psaltis A.J., Wormald P.J., Vreugde S. (2023). Pharokka: A fast scalable bacteriophage annotation tool. Bioinformatics.

[65] Terzian P., Ndela E.O., Galiez C., Lossouarn J., Bucio R.E.P., Mom R., Toussaint A., Petit M.-A., Enault F. (2021). PHROG: Families of prokaryotic virus proteins clustered using remote homology. NAR Genom. Bioinform..

[66] Nishimura Y., Yoshida T., Kuronishi M., Uehara H., Ogata H., Goto S. (2017). ViPTree: The viral proteomic tree server. Bioinformatics.

[67] Hyman P. (2019). Phages for Phage Therapy: Isolation, Characterization, and Host Range Breadth. Pharmaceuticals.

[68] Mourosi J.T., Awe A., Guo W., Batra H., Ganesh H. (2022). Understanding Bacteriophage Tail Fiber Interaction with Host Surface Receptor: The Key “Blueprint” for Reprogramming Phage Host Range. Int. J. Mol. Sci..

[69] Olkkonen V.M., Bamford D.H. (1989). Quantitation of the adsorption and penetration stages of bacteriophage phi 6 infection. Virology.

[70] Harada L.K., Silva E.C., Rossi F.P.N., Cieza B., Oliveira T.J., Pereira C., Tomazetto G., Silva B.B., Squina F.M., Vila M.M.D.C. (2022). Characterization and in vitro testing of newly isolated lytic bacteriophages for the biocontrol of *Pseudomonas aeruginosa*. Future Microbiol..

[71] Payne R., Jansen V. (2001). Understanding bacteriophage therapy as a density-dependent kinetic process. J. Theor. Biol..

[72] Abedon S.T. (2011). Lysis from without. Bacteriophage.

[73] Shao Y., Wang I.-N. (2008). Bacteriophage adsorption rate and optimal lysis time. Genetics.

[74] Jones J.B., Jackson L.E., Balogh B., Obradovic A., Iriarte F.B., Momol M.T. (2007). Bacteriophages for plant disease control. Annu. Rev. Phytopathol..

[75] Balogh B., Jones J.B., Iriarte F.B., Momol M.T. (2010). Phage therapy for plant disease control. Curr. Pharm. Biotechnol..

[76] Abedon S., Culler R.R. (2007). Optimizing bacteriophage plaque fecundity. J. Theor. Biol..

[77] Balcão V.M., Basu A., Cieza B., Rossi F.N., Pereira C., Vila M.M.D.C., Setubal J.C., Ha T., da Silva A.M. (2022). *Pseudomonas*-tailed lytic phages: Genome mechanical analysis and putative correlation with virion morphogenesis yield. Future Microbiol..

[78] Mateus L., Costa L., Silva Y.J., Pereira C., Cunha A., Almeida A. (2014). Efficiency of phage cocktails in the inactivation of *Vibrio* in aquaculture. Aquaculture.

[79] Pereira C., Moreirinha C., Lewicka M., Almeida P., Clemente C., Cunha Â., Delgadillo I., Romalde J., Nunes M.L., Almeida A. (2016). Bacteriophages with potential to inactivate *Salmonella* Typhimurium: Use of single phage suspensions and phage cocktails. Virus Res..

[80] Gill J.J., Hyman P. (2010). Phage choice, isolation, and preparation for phage therapy. Curr. Pharm. Biotechnol..

[81] Pinheiro L., Pereira C., Frazão C., Balcão V., Almeida A. (2019). Efficiency of phage φ6 for biocontrol of *Pseudomonas syringae* pv. *syringae*: An in vitro preliminary study. Microorganisms.

[82] Duarte J., Pereira C., Moreirinha C., Salvio R., Lopes A., Wang D., Almeida A. (2018). New insights on phage efficacy to control *Aeromonas salmonicida* in aquaculture systems: An in vitro preliminary study. Aquaculture.

[83] Forti F., Roach D.R., Cafora M., Pasini M.E., Horner D.S., Fiscarelli E.V., Rossitto M., Cariani L., Briani F., Debarbieux L. (2018). Design of a Broad-Range Bacteriophage Cocktail That Reduces *Pseudomonas aeruginosa* Biofilms and Treats Acute Infections in Two Animal Models. Antimicrob. Agents Chemother..

[84] Costa P., Pereira C., Gomes A., Almeida A. (2019). Efficiency of single phage suspensions and phage cocktail in the inactivation of Escherichia coli and *Salmonella* Typhimurium: An in vitro preliminary study. Microorganisms.

[85] Silva E.C., Rodrigues L.M.R., Vila M.M.D.C., Balcão V.M. (2023). Newly isolated phages preying on *Pseudomonas syringae* pv. *garcae*: In vitro and ex vivo inactivation studies in coffee plant leafs. Enzyme Microb. Technol..

[86] Wright R.C., Friman V.-P., Smith M.C.M., Brockhurst M.A. (2018). Cross-resistance is modular in bacteria–phage interactions. PLoS Biol..

[87] Rombouts S., Volckaert A., Venneman S., Declercq B., Vandenheuvel D., Allonsius C.N., Van Malderghem C., Jang H.B., Briers Y., Noben J.P. (2016). Characterization of novel bacteriophages for biocontrol of bacterial blight in leek caused by *Pseudomonas syringae* pv. *porri*. Front. Microbiol..

[88] Li M., Jin Y., Lin H., Wang J., Jiang X. (2018). Complete genome of a novel lytic *Vibrio parahaemolyticus* phage VPp1 and characterization of its endolysin for antibacterial activities. J. Food Prot..

[89] Ferran A.A., Lacroix M.Z., Gourbeyre O., Huesca A., Gaborieau B., Debarbieux L., Bousquet-Mélou A. (2022). The selection of antibiotic- and bacteriophage-resistant *Pseudomonas aeruginosa* is prevented by their combination. Microbiol. Spectr..

[90] Buttimer C., McAuliffe O., Ross R.P., Hill C., O’Mahony J., Coffey A. (2017). Bacteriophages and bacterial plant diseases. Front. Microbiol..

[91] Labrie S.J., Samson J.E., Moineau S. (2010). Bacteriophage resistance mechanisms. Nat. Rev. Microbiol..

[92] Koskella B., Brockhurst M.A. (2014). Bacteria-phage coevolution as a driver of ecological and evolutionary processes in microbial communities. FEMS Microbiol. Rev..

[93] Frampton R.A., Pitman A.R., Fineran P.C. (2012). Advances in bacteriophage-mediated control of plant pathogens. Int. J. Microbiol..

[94] Chan B.K., Abedon S.T., Loc-Carrillo C. (2013). Phage cocktails and the future of phage therapy. Future Microbiol..

[95] Torres-Barceló C. (2018). The disparate effects of bacteriophages on antibiotic-resistant bacteria. Emerg. Microbes Infect..

[96] Svircev A., Roach D., Castle A. (2018). Framing the future with bacteriophages in agriculture. Viruses.

[97] Cutler T., Zimmerman J. (2011). Ultraviolet irradiation and the mechanisms underlying its inactivation of infectious agents. Anim. Heal. Res. Rev..

[98] Wurtmann E.J., Wolin S.L. (2009). RNA under attack: Cellular handling of RNA damage. Crit. Rev. Biochem. Mol. Biol..

[99] Manjula B., Varaprasad K., Sadiku R., Raju K.M. (2013). Preparation and characterization of sodium alginate-based hydrogels and their in vitro release studies. Adv. Polym. Technol..

[100] Rios A., Vila M., Lima R., Del Fiol F., Tubino M., Teixeira J., Balcão V. (2018). Structural and functional stabilization of bacteriophage particles within the aqueous core of a W/O/W multiple emulsion: A potential biotherapeutic system for the inhalational treatment of bacterial pneumonia. Process Biochem..

[101] Balcão V.M., Barreira S.V.P., Nunes T.M., Chaud M.V., Tubino M., Vila M.M.D.C. (2014). Carbohydrate hydrogels with stabilized phage particles for bacterial biosensing: Bacterium diffusion studies. Appl. Biochem. Biotechnol..

[102] Balcão V.M., Vila M.M.D.C. (2015). Structural and functional stabilization of protein entities: State-of-the-art. Adv. Drug Deliv. Rev..

[103] Balcão V.M., Glasser C.A., Chaud M.V., del Fiol F.S., Tubino M., Vila M.M.D.C. (2014). Biomimetic aqueous-core lipid nanoballoons integrating a multiple emulsion formulation: A suitable housing system for viable lytic bacteriophages. Colloids Surf. B Biointerfaces.

[104] Hsu C.-H., Lo C.-Y., Liu J.-K., Lin C.-S. (2000). Control of the eel (*Anguilla japonica*) pathogens, *Aeromonas hydrophila* and *Edwardsiella tarda*, by bacteriophages. J. Fish. Soc. Taiwan.

[105] Pasharawipas T., Manopvisetcharean J., Flegel T. (2011). Phage treatment of *Vibrio harveyi*: A general concept of protection against bacterial infection. Res. J. Microbiol..

[106] Prasad Y., Arpana, Kumar D., Sharma A. (2011). Lytic bacteriophages specific to *Flavobacterium columnare* rescue catfish, *Clarias batrachus* (Linn.) from columnaris disease. J. Environ. Biol..

[107] Oechslin F. (2018). Resistance development to bacteriophages occurring during bacteriophage therapy. Viruses.

[108] Kasurinen J., Spruit C.M., Wicklund A., Pajunen M.I., Skurnik M. (2021). Screening of Bacteriophage Encoded Toxic Proteins with a Next Generation Sequencing-Based Assay. Viruses.

[109] Kharina A.V., Zaika S.A., Yumyna Y.M., Zelena P.P., Kornienko N.O., Kosenko Y.A., Polischuk V.P. (2015). Detection of *Proteus mirabilis* and *Enterobacter cloacae* in tomatto and pepper fruits and isolation of their bacteriophages. Sci. Rep. Natl. Univ. Life Resour. Environ. Manag. Ukr..

[110] Oliveira H., Sillankorva S., Merabishvili M., Kluskens L.D., Azeredo J. (2015). Unexploited opportunities for phage therapy. Front. Pharmacol..

[111] Harrison E., Brockhurst M.A. (2017). Ecological and Evolutionary Benefits of Temperate Phage: What Does or Doesn’t Kill You Makes You Stronger. BioEssays.

