# Supplementary material for: Three Novel Bacteriophages for the Biocontrol of Pseudomonas syringae pv. actinidiae on Artificially Contaminated Kiwifruit Leaves
Source: Pathogens. 2025 Dec 5;14(12):1247. doi: 10.3390/pathogens14121247 (PMC12736001; doi:10.3390/pathogens14121247)
Supplement: Supplementary file 1 [file pathogens-14-01247-s001.zip › pathogens-4030495-supplementary.pdf]

Table S1. Coding sequences identified in PSA-2T phage.

Gene	Start	Stop	Frame	Region	Putative Function	PHROGs Category
FLBKAEYF_0001	1	1266	+	CDS	hypothetical protein	unknown function
FLBKAEYF_0002	1263	3065	+	CDS	portal protein	head and packaging
FLBKAEYF_0003	3062	3235	+	CDS	DNA ligase	DNA, RNA and nucleotide metabolism
FLBKAEYF_0004	3238	4365	+	CDS	head maturation protease	head and packaging
FLBKAEYF_0005	4375	4851	+	CDS	virion structural protein	head and packaging
FLBKAEYF_0006	4853	5812	+	CDS	major head protein	head and packaging
FLBKAEYF_0007	5823	6206	+	CDS	hypothetical protein	unknown function
FLBKAEYF_0008	6261	6419	+	CDS	hypothetical protein	unknown function
FLBKAEYF_0009	6456	6977	+	CDS	head-tail adaptor Ad1	connector
FLBKAEYF_0010	6974	7969	+	CDS	head morphogenesis	head and packaging
FLBKAEYF_0011	7979	8362	+	CDS	hypothetical protein	unknown function
FLBKAEYF_0012	8362	8739	+	CDS	tail completion or Neck1 protein	connector
FLBKAEYF_0013	8736	9131	+	CDS	tail terminator	connector
FLBKAEYF_0014	9196	10116	+	CDS	minor tail protein	tail
FLBKAEYF_0015	10188	10661	+	CDS	hypothetical protein	unknown function
FLBKAEYF_0016	10679	10930	+	CDS	hypothetical protein	unknown function
FLBKAEYF_0017	11098	11331	+	CDS	hypothetical protein	unknown function
FLBKAEYF_0018	12756	15428	+	CDS	tail length tape measure protein	tail
FLBKAEYF_0019	15446	16030	+	CDS	distal tail protein	tail
FLBKAEYF_0020	16030	16632	+	CDS	tail protein	tail
FLBKAEYF_0021	16575	17033	+	CDS	hypothetical protein	unknown function
FLBKAEYF_0022	17033	20170	+	CDS	central tail fiber J	tail
FLBKAEYF_0023	20167	20535	+	CDS	hypothetical protein	unknown function
FLBKAEYF_0024	20532	21194	+	CDS	hypothetical protein	unknown function
FLBKAEYF_0025	21223	22335	+	CDS	hypothetical protein	unknown function
FLBKAEYF_0026	23078	22401	-	CDS	hypothetical protein	unknown function
FLBKAEYF_0027	23112	23645	+	CDS	endolysin	lysis
FLBKAEYF_0028	23642	24157	+	CDS	Rz-like spanin	lysis
FLBKAEYF_0029	24170	24382	+	CDS	hypothetical protein	unknown function
FLBKAEYF_0030	24379	25623	+	CDS	lipase	moron, auxiliary metabolic gene and host takeover
FLBKAEYF_0031	27129	25945	-	CDS	integrase	integration and excision
FLBKAEYF_0032	27776	27453	-	CDS	hypothetical protein	unknown function
FLBKAEYF_0033	28411	27773	-	CDS	hypothetical protein	unknown function
FLBKAEYF_0034	28722	28408	-	CDS	hypothetical protein	unknown function
FLBKAEYF_0035	29036	28785	-	CDS	hypothetical protein	unknown function
FLBKAEYF_0036	30718	29087	-	CDS	DNA methyltransferase	other
FLBKAEYF_0037	31253	30789	-	CDS	Lar-like restriction alleviation protein	moron, auxiliary metabolic gene and host takeover
FLBKAEYF_0038	31465	31250	-	CDS	hypothetical protein	unknown function
FLBKAEYF_0039	32001	31462	-	CDS	hypothetical protein	unknown function
FLBKAEYF_0040	32168	31998	-	CDS	hypothetical protein	unknown function
FLBKAEYF_0041	33465	32224	-	CDS	DNA methyltransferase	other
FLBKAEYF_0042	33834	33523	-	CDS	hypothetical protein	unknown function
FLBKAEYF_0043	34172	33825	-	CDS	hypothetical protein	unknown function
FLBKAEYF_0044	34448	34260	-	CDS	hypothetical protein	unknown function
FLBKAEYF_0045	35166	34495	-	CDS	DNA polymerase exonuclease subunit	DNA, RNA and nucleotide metabolism
FLBKAEYF_0046	35853	35197	-	CDS	single strand DNA binding protein	DNA, RNA and nucleotide metabolism
FLBKAEYF_0047	36503	35865	-	CDS	Sak4-like ssDNA annealing protein	DNA, RNA and nucleotide metabolism

FLBKAEYF_0048	37790	36666	-	CDS	hypothetical protein	unknown function
FLBKAEYF_0049	38129	37959	-	CDS	hypothetical protein	unknown function
FLBKAEYF_0050	38377	38126	-	CDS	hypothetical protein	unknown function
FLBKAEYF_0051	38616	38374	-	CDS	hypothetical protein	unknown function
FLBKAEYF_0052	39464	38637	-	CDS	hypothetical protein	unknown function
FLBKAEYF_0053	39798	39673	-	CDS	hypothetical protein	unknown function
FLBKAEYF_0054	40444	39923	-	CDS	hypothetical protein	unknown function
FLBKAEYF_0055	41732	40950	-	CDS	CI-like repressor	transcription regulation
FLBKAEYF_0056	41773	42000	+	CDS	transcriptional repressor	transcription regulation
FLBKAEYF_0057	42032	42238	+	CDS	hypothetical protein	unknown function
FLBKAEYF_0058	42213	42482	+	CDS	hypothetical protein	unknown function
FLBKAEYF_0059	42560	43294	+	CDS	anti-repressor Ant	transcription regulation
FLBKAEYF_0060	43381	43746	+	CDS	hypothetical protein	unknown function
FLBKAEYF_0061	43746	44540	+	CDS	replication initiation protein	DNA, RNA and nucleotide metabolism
FLBKAEYF_0062	44530	45348	+	CDS	DnaC-like helicase loader	DNA, RNA and nucleotide metabolism
FLBKAEYF_0063	45348	45503	+	CDS	hypothetical protein	unknown function
FLBKAEYF_0064	45500	45688	+	CDS	hypothetical protein	unknown function
FLBKAEYF_0065	45685	46008	+	CDS	hypothetical protein	unknown function
FLBKAEYF_0066	46005	46739	+	CDS	hypothetical protein	unknown function
FLBKAEYF_0067	46736	46837	+	CDS	hypothetical protein	unknown function
FLBKAEYF_0068	46830	47228	+	CDS	NinB/ Orf homologous recombination mediator	DNA, RNA and nucleotide metabolism
FLBKAEYF_0069	47228	47395	+	CDS	hypothetical protein	unknown function
FLBKAEYF_0070	47392	47976	+	CDS	NinG/ Rap DNA junction specific endonuclease	DNA, RNA and nucleotide metabolism
FLBKAEYF_0071	47973	48554	+	CDS	hypothetical protein	unknown function
FLBKAEYF_0072	48781	49152	+	CDS	hol-like chemotaxis	other
FLBKAEYF_0073	49152	49490	+	CDS	hypothetical protein	unknown function
FLBKAEYF_0074	49538	49789	+	CDS	hypothetical protein	unknown function
FLBKAEYF_0075	49786	49920	+	CDS	hypothetical protein	unknown function
FLBKAEYF_0076	49925	50548	+	CDS	transposase	integration and excision
FLBKAEYF_0077	50580	51089	+	CDS	hypothetical protein	unknown function

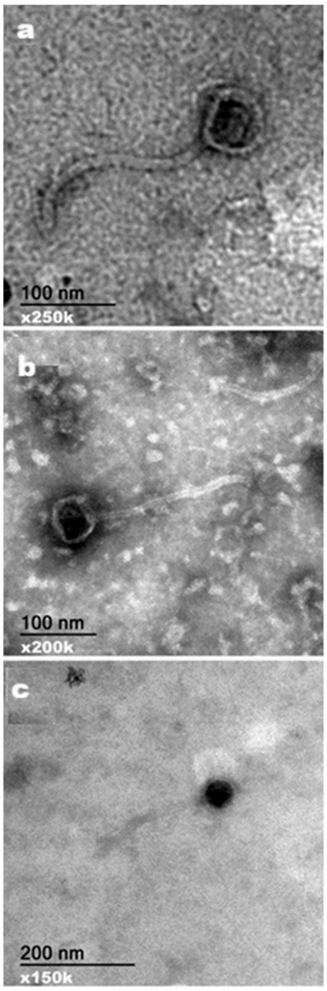

Figure S1. Transmission electron photomicrographs of isolated PSA phages. (**a:** phage PSA-2T (x250k magnification); **b:** phage PSA-6F (x200k magnification); **c:** phage PSA-7F (x150k magnification)).

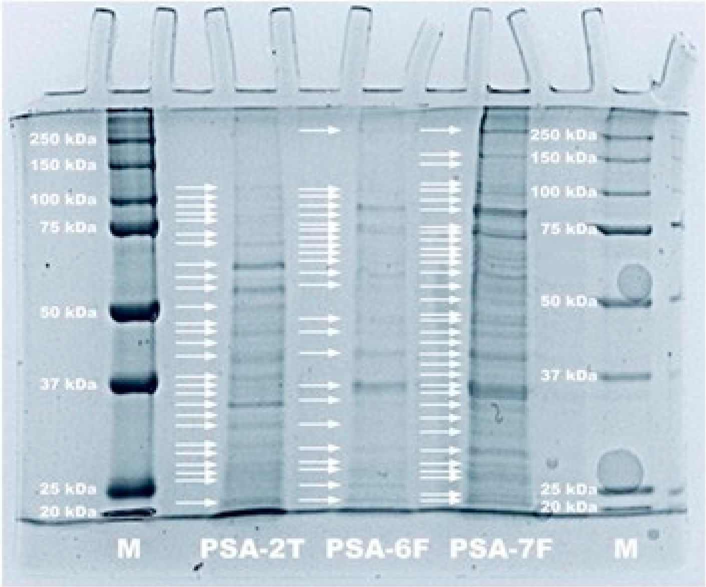

Figure S2. Coomassie-stained electrophoretogram of the structural proteins of phages PSA-2T, PSA-6F and PSA-7F and wide-range molecular weight markers (lanes M).

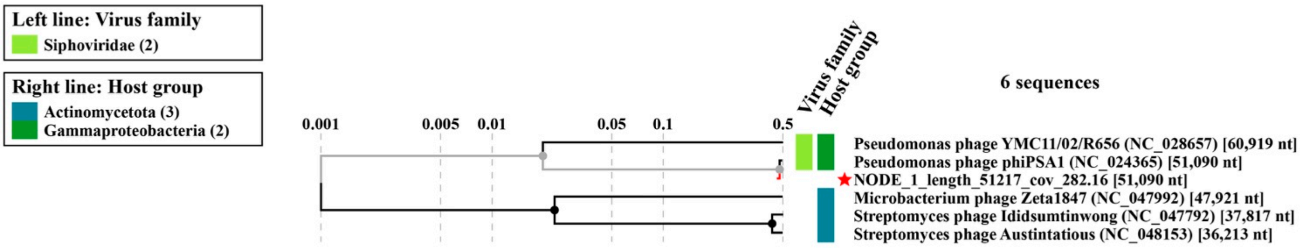

Figure S3. Viral proteomic tree from ViPTree analysis of phage PSA-2T and related phages. 5633 phage genomes were used as reference sequences to build phylogenetic trees using ViPTree. Expanded view of the region of the tree containing the most closely related phages. The branch containing phage PSA-2T is displayed in red. Branch length indicates evolutionary distance. The colored bars represent the virus family (left bar) and host group (right bar). The red star pinpoints the location of phage PSA-2T.

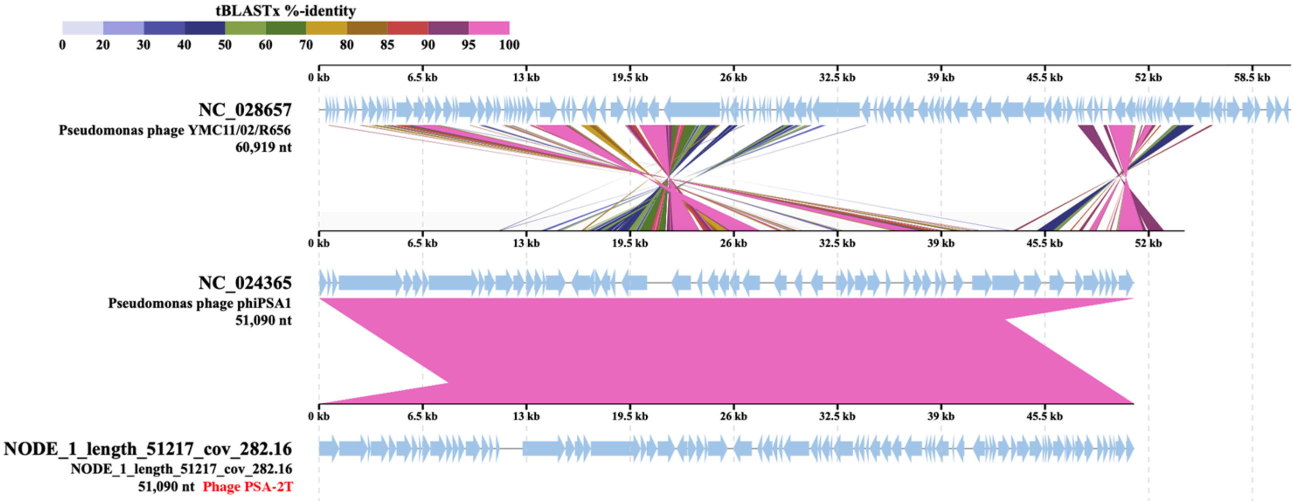

Figure S4. Genome sequence alignment and comparison of phage PSA-2T with two other phage genomes exhibiting co-linearity detected by TBLASTX tool from BLAST+ v2.13.0 using the ViPTree server [66]. Homologous regions detected by a TBLASTX search are connected by segments colored based on amino acid identity. The upper color bar shows the % identity of TBLASTX.
